# Supplementary material for: POSNOC—POsitive Sentinel NOde: adjuvant therapy alone versus adjuvant therapy plus Clearance or axillary radiotherapy: a randomised controlled trial of axillary treatment in women with early-stage breast cancer who have metastases in one or two sentinel nodes
Source: BMJ Open. 2021 Dec 2;11(12):e054365. doi: 10.1136/bmjopen-2021-054365 (PMC8640630; doi:10.1136/bmjopen-2021-054365)
Supplement: Author's manuscript [file bmjopen-2021-054365.draft_revisions.pdf]

BMJ Open is committed to open peer review. As part of this commitment we make the peer review history of every article we publish publicly available.

When an article is published we post the peer reviewers' comments and the authors' responses online. We also post the versions of the paper that were used during peer review. These are the versions that the peer review comments apply to.

The versions of the paper that follow are the versions that were submitted during the peer review process. They are not the versions of record or the final published versions. They should not be cited or distributed as the published version of this manuscript.

BMJ Open is an open access journal and the full, final, typeset and author-corrected version of record of the manuscript is available on our site with no access controls, subscription charges or pay-per-view fees (http://bmjopen.bmj.com).

If you have any questions on BMJ Open's open peer review process please email info.bmjopen@bmj.com

BMJ Open

POSNOC - Positive Sentinel Node: adjuvant therapy alone versus adjuvant therapy plus Clearance or axillary radiotherapy. A randomised controlled trial of axillary treatment in women with early stage breast cancer who have metastases in one or two sentinel nodes

Journal:	*BMJ Open*
Manuscript ID	bmjopen-2021-054365
Article Type:	Protocol
Date Submitted by the Author:	09-Jun-2021

Complete List of Authors:	Goyal, Amit; Royal Derby Hospital,<br>Mann, G. Bruce; The University of Melbourne<br>Fallowfield, Lesley; University of Sussex, SHORE-C, Brighton & Sussex Medical School<br>Duley, Lelia; Nottingham Clinical Trials Unit, University of Nottingham<br>Reed, Malcolm; Brighton and Sussex Medical School,<br>Dodwell, David; Oxford University, Nuffield Department of Population Health<br>Coleman, Robert E.; Weston Park Hospital<br>Fakis, Apostolos; Royal Derby Hospital, Research and Development<br>Newcombe, Robert; Cardiff University, Department of Primary Care and Public Health<br>Jenkins, Valerie; University of Sussex,<br>Whitham, Diane; The University of Nottingham, Clinical Trials Unit<br>Childs, Margo; University of Nottingham,<br>Whynes, David; University of Nottingham<br>Keeley, Vaughn; University of Nottingham,<br>Ellis, Ian ; University of Nottingham, Division of Oncology, School of Medicine<br>Fairbrother, Patricia; Independent Cancer Patients' Voice,<br>Sadiq, Shabina; Nottingham Clinical Trials Unit, University of Nottingham<br>Monson, Kathryn; Brighton & Sussex Medical School, Sussex Health Outcomes, Research & Education in Cancer (SHORE-C)<br>Montgomery, Alan; Nottingham Clinical Trials Unit, University of Nottingham<br>Tan, Wei; University of Nottingham School of Medecine, Nottingham Clinical Trials Unit<br>Vale, Luke; Newcastle University, Health Economics Group, Population Health Sciences Institute<br>Homer, Tara; Newcastle University, Health Economics Group, Population Health Sciences Institute<br>Badger, Heath; Breast Cancer Trials<br>Haines, Rachel; University of Nottingham School of Medecine, Nottingham Clinical Trials Unit<br>Lewis, Mickey; Nottingham Clinical Trials Unit, University of Nottingham<br>Megias, Daniel; National Radiotherapy Trials Quality Assurance Group, Mount Vernon Cancer Centre

	Nabi, Zohal; National Radiotherapy Trials Quality Assurance Group, Mount Vernon Cancer Centre<br>Singh, Preetinder; National Radiotherapy Trials Quality Assurance Group, Mount Vernon Cancer Centre<br>Caraman, Andrei; National Radiotherapy Trials Quality Assurance Group, Mount Vernon Cancer Centre<br>Miles, Elizabeth; National Radiotherapy Trials Quality Assurance Group, Mount Vernon Hospital

Keywords:	Breast surgery < SURGERY, Breast tumours < ONCOLOGY, Adult radiotherapy < RADIOTHERAPY

SCHOLARONE™ Manuscripts

I, the Submitting Author has the right to grant and does grant on behalf of all authors of the Work (as defined in the below author licence), an exclusive licence and/or a non-exclusive licence for contributions from authors who are: i) UK Crown employees; ii) where BMJ has agreed a CC-BY licence shall apply, and/or iii) in accordance with the terms applicable for US Federal Government officers or employees acting as part of their official duties; on a worldwide, perpetual, irrevocable, royalty-free basis to BMJ Publishing Group Ltd ("BMJ") its licensees and where the relevant Journal is co-owned by BMJ to the co-owners of the Journal, to publish the Work in this journal and any other BMJ products and to exploit all rights, as set out in our licence.

The Submitting Author accepts and understands that any supply made under these terms is made by BMJ to the Submitting Author unless you are acting as an employee on behalf of your employer or a postgraduate student of an affiliated institution which is paying any applicable article publishing charge ("APC") for Open Access articles. Where the Submitting Author wishes to make the Work available on an Open Access basis (and intends to pay the relevant APC), the terms of reuse of such Open Access shall be governed by a Creative Commons licence – details of these licences and which Creative Commons licence will apply to this Work are set out in our licence referred to above.

Other than as permitted in any relevant BMJ Author's Self Archiving Policies, I confirm this Work has not been accepted for publication elsewhere, is not being considered for publication elsewhere and does not duplicate material already published. I confirm all authors consent to publication of this Work and authorise the granting of this licence.

For peer review only

Title: POSNOC - POsitive Sentinel NOde: adjuvant therapy alone versus adjuvant therapy plus Clearance or axillary radiotherapy. A randomised controlled trial of axillary treatment in women with early stage breast cancer who have metastases in one or two sentinel nodes

Authors: Amit Goyal[1], G Bruce Mann[2], Lesley Fallowfield[3], Lelia Duley[4], Malcolm W Reed[5], David Dodwell[6], Robert Coleman[7], Apostolos Fakis[1], Robert G Newcombe[8], Valerie Jenkins[3], Diane Whitham[4], Margaret Childs[4], David Whynes[4], Vaughan Keeley[1], Ian Ellis[9], Patricia Fairbrother[10], Shabina Sadiq[4], Kathryn Monson[3], Alan A Montgomery[4], Wei Tan[4], Luke Vale[11], Tara Homer[11], Heath Badger[12], Rachel Haines[4], Mickey Lewis[4], Daniel Megias[13], Zohal Nabi[13], Preetinder Singh[13], Andrei Caraman[13], Elizabeth Miles[13] on behalf of the POSNOC Trial Management Group

Institutions: [1]University Hospitals of Derby and Burton NHS Foundation Trust, Derby, UK; [2]Victorian Comprehensive Cancer Centre, Melbourne, Australia; [3]SHORE-C, University of Sussex, Brighton, UK; [4]Nottingham Clinical Trials Unit, University of Nottingham, Nottingham, UK; [5]Brighton and Sussex Medical School, University of Sussex, Brighton, UK; [6]Nuffield Department of Population Health, University of Oxford, Oxford, UK; [7]Weston Park Hospital, Sheffield, UK; [8]Cardiff University, Cardiff, UK; [9]University of Nottingham, Nottingham, UK; [10]Independent Cancer Patients' Voice, London, UK; [11]Newcastle University, Newcastle, UK; [12]Breast Cancer Trials, Newcastle, Australia; [13]National Radiotherapy Trials QA (RTTQA) Group, Mount Vernon Cancer Centre, Northwood, UK

Correspondence: Amit Goyal, University Hospitals of Derby and Burton NHS Foundation Trust, Derby, UK. Email: amit.goyal@nhs.net

Tel: +44(0)1332 785538, Twitter handle: @_amit_goyal

Word count: 4000

Figures: 1

Tables: 1

Abstract

Introduction

ACOSOG-Z0011(Z11) trial showed that axillary node clearance(ANC) may be omitted in women with ≤2 positive nodes undergoing breast conserving surgery (BCS) and whole breast radiotherapy (RT). A confirmatory study is needed to clarify the role of axillary treatment in women with ≤2 macrometastases undergoing BCS and groups that were not included in Z11 e.g. mastectomy and those with microscopic extranodal invasion.

The primary objective of POSNOC is to evaluate whether for women with breast cancer and 1 or 2 macrometastases, adjuvant therapy alone is non-inferior to adjuvant therapy plus axillary treatment, in terms of 5-year axillary recurrence.

Methods and analysis

POSNOC is a pragmatic, multicentre, non-inferiority, international trial with participants randomised in a 1:1 ratio. Women are eligible if they have T1/T2, unifocal or multifocal invasive breast cancer, and 1 or 2 macrometastases at sentinel node biopsy, with or without extranodal extension. In the intervention group women receive adjuvant therapy alone, in the standard care group they receive ANC or axillary RT. In both groups women receive adjuvant therapy, according to local guidelines. This includes systemic therapy and, if indicated, RT to breast or chest wall. The UK Radiotherapy Trials Quality Assurance group manages the in-built Radiotherapy Quality Assurance programme. Primary endpoint is 5-year axillary recurrence. Secondary outcomes are arm morbidity assessed by LBCQ and QuickDASH questionnaires; Quality of Life and anxiety as assessed with FACT B+4 and STAI questionnaires respectively; other oncological outcomes; economic evaluation using EQ-5D-5L. Target sample size is 1900. Primary analysis is per protocol. Recruitment started on 1 August 2014 and as of 09 June 2021, 1866 participants have been randomised.

Ethics and dissemination: Protocol was approved by the National Research Ethics Service Committee East Midlands – Nottingham 2 (REC reference: 13/EM/0459). Results will be submitted for publication in peer-reviewed journals.

Trial registration number: ISRCTN54765244; NCT02401685

Strengths and limitations of this study

- POSNOC includes women undergoing mastectomy and those with extranodal invasion
- Only women with 1 or 2 macrometastases are eligible for randomisation

For peer review only

- POSNOC has an in-built radiotherapy quality assurance programme co-ordinated by the UK Radiotherapy Trials QA (RTTQA) Group
- POSNOC is an international trial - UK, Australia and New Zealand

Key words: POSNOC, sentinel node biopsy, breast cancer, axillary node clearance, axillary radiotherapy

For peer review only

Introduction

Currently, women with early breast cancer and 1 or 2 macrometastases in sentinel lymph nodes (SNs) generally undergo some form of axillary treatment, either surgical axillary node clearance (ANC) or radiotherapy to the axilla (ART). The use of axillary treatment is based on the assumption that it reduces the risk of axillary recurrence and might improve survival. However, axillary treatment damages lymphatic drainage from the arm and women can subsequently develop lymphoedema, restricted shoulder movement, pain, numbness, and other sensory problems. These adverse effects interfere with daily activities, are distressing, impair quality of life (QoL) and are costly to the NHS in terms of rehabilitative treatments, as they are often permanent and symptom relief is difficult.

Axillary treatment may no longer be necessary for this group of women, as they usually have a low axillary tumour burden and current systemic therapy is effective at preventing breast and axillary recurrence[1]. Also, sentinel node biopsy has already removed the lymph nodes most likely to have metastasis[2]. Moreover, when adjuvant therapy includes radiotherapy to the breast or chest wall, the lower axilla may be treated as it is often included in the tangential irradiation field, and some lower level axillary nodes may be removed at mastectomy[3].

This hypothesis is supported by the Z11 trial[4] that showed that ANC may be omitted in women with ≤2 positive nodes undergoing breast conserving surgery (BCS) and receiving whole breast RT. Axillary recurrence was low, and there were no clear differences between the two groups (ANC 0.5% vs. no ANC 0.9%) at 6.3 years. Z11 has not changed clinical practice because of several limitations. The study did not meet its recruitment goal, approximately 40% of participants had micrometastases, many were lost to follow-up (19.4%), and there was a lack of radiation therapy quality assurance. Most US radiation oncologists continue to treat the undissected axilla in women with micro or macro metastases with ART[5]. Therefore, it appears that clinicians in the US are interpreting Z11 in line with AMAROS study which showed that ART is a less morbid alterative to axillary lymph node dissection (ALND) in women with low volume nodal disease[6] rather than omitting axillary treatment. Furthermore, the results are not applicable to subgroups that were not included such as those with mastectomy and microscopic extranodal invasion.

For peer review only

Objective

The primary objective of the POSNOC trial is to evaluate whether, for women with early breast cancer and one or two nodes with macrometastases, adjuvant therapy alone is non-inferior to adjuvant therapy plus axillary treatment (ANC or ART), in terms of 5-year axillary recurrence.

Methods and analysis

Design: POSNOC is a pragmatic randomised, multicentre, non-inferiority, international (UK, Australia, New Zealand) trial. Target sample size is 1900 participants, with 5-year follow-up. Flow of participants through the trial is summarised in Figure 1.

Randomisation: Participants are allocated via a secure internet-based system, using minimisation that is weighted toward minimising imbalance between trial arms with probability 0.8. Allocation is stratified by recruiting site and minimised by the following variables: age (<50, ≥50 years); type of breast surgery (breast-conserving surgery (BCS), mastectomy); estrogen receptor status (ER) (positive, negative); number of positive nodes (1, 2); intra-operative sentinel assessment using OSNA (yes, no).

Target population: Women with unifocal or multifocal invasive breast cancer with the largest lesion ≤5cm, who have 1 or 2 sentinel nodes with macrometastases (>2mm). Women are usually screened for eligibility after their initial breast surgery and sentinel node biopsy. In hospitals where sentinel node histology is assessed intra-operatively, women are asked to give their consent pre-operatively and then randomised intra-operatively if their sentinel nodes are found to be positive.

Inclusion Criteria: Women are eligible for inclusion if ALL of the following criteria apply:

- 18 years or older
- Unifocal or multi-focal invasive tumour ≤5 cm in its largest dimension, measured pathologically or largest invasive tumour diameter on radiology for women who are randomised intra-operatively or who undergo sentinel node biopsy before neoadjuvant therapy (tumour size is based only on the single largest tumour)
- At sentinel node biopsy have 1 or 2 nodes with macrometastases (tumour deposit >2.0mm in largest dimension or defined as macrometastases on molecular assay)
- Fit for axillary treatment and adjuvant therapy
- Have given informed consent

Exclusion Criteria: Women are excluded if they have any of the following:

- Bilateral invasive breast cancer

For peer review only

- more than 2 nodes with macrometastases
- neoadjuvant therapy for breast cancer except
  - o if sentinel node biopsy performed prior to neoadjuvant therapy in women with early breast cancer
  - o short duration of neoadjuvant endocrine therapy (up to 3-months)
- previous axillary surgery on the same body side as the scheduled sentinel node biopsy
- not planned to receive adjuvant systemic therapy
- previous cancer less than 5-years previously or concomitant malignancy except
  - o basal or squamous cell carcinoma of the skin
  - o in situ carcinoma of the cervix
  - o in situ melanoma
  - o contra- or ipsilateral in situ breast cancer

Trial treatment arms: All participants receive adjuvant therapy according to local guidelines. This includes chemotherapy and/or endocrine therapy for all women, and radiotherapy to breast or chest wall if indicated. Human epidermal growth factor receptor 2 (HER2) targeted treatment is administered when indicated.

Trial participants are allocated to one of the following two groups:

(i) Adjuvant therapy alone (intervention group)

See above for adjuvant therapy. Axillary and supraclavicular fossa radiotherapy is not allowed.

(ii) Adjuvant therapy plus axillary treatment (standard care group)

See above for adjuvant therapy. Axillary treatment can be axillary node clearance or axillary radiotherapy as per local guidelines.

Outcomes

Primary Outcome: The primary outcome is 5-year axillary recurrence defined as pathologically (cytology or biopsy) and/or radiologically confirmed recurrence in lymph nodes draining the primary tumour site.

Secondary Outcomes:

- Arm morbidity assessed by a) Lymphoedema and Breast Cancer Questionnaire (LBCQ)[7] - the definition of lymphoedema is 'yes' to the two questions -'heaviness during the past year' and 'swelling now'; b) QuickDASH questionnaire[8] - Shoulder, arm and hand disability is defined as a change from baseline in the QuickDASH score of at least 14 points.

For peer review only

- QoL assessed by Functional Assessment of Cancer Therapy-Breast+4 (FACT B+4) questionnaire[9]. The total FACT B+4 score reflects global QoL and comprises physical (7 items), functional (7 items), social (7 items) and emotional well-being (6 items) plus the breast cancer concerns and arm morbidity scale (14 items) (total of 41 items; total score ranges from 0-164). The 5 item arm morbidity subscale score comprises the sum of the scores from items B3, B10, B11, B12, B13 (range 0-20) and will be analysed separately. Analyses will include the proportion of participants in the two allocated treatment groups reporting 'somewhat', 'quite a bit' and 'very much' for each of the 5 items.
- Anxiety assessed by using the Spielberger State/Trait Anxiety Inventory(STAI)[10]. The STAI consists of 2 questionnaires with 20 items. It assesses anxiety proneness (Trait) and the current state of anxiety or anxiety change (State). Each item is rated on a four point Likert scale. High STAI scores signify greater anxiety. The Trait anxiety is measured only once and the State at each time point.
- Economic outcomes will include costs to the NHS, quality-adjusted life years (QALYs) based on responses to the EQ-5D-5L, and the primary outcome, axillary recurrence. For more details, see Economic Analysis section below.
- Local (breast or chest wall) recurrence; defined as pathologically (cytology or biopsy) and/or radiologically confirmed recurrence after mastectomy in the skin or soft tissue of the chest wall within the anatomical area bounded by the mid-sternal line, the clavicle, the posterior axillary line and the costal margin or any type of breast carcinoma in the breast after conservation therapy.
- Regional (nodal) recurrence; defined as pathologically (cytology or biopsy) and/or radiologically confirmed recurrent tumour in the lymph nodes in the ipsilateral axilla, infraclavicular, supraclavicular fossa, interpectoral area or ipsilateral internal mammary chain.
- Distant metastasis; defined as confirmed metastasis (positive pathology and/or definitive evidence on imaging) in all other sites of recurrence and may include those classified as: soft-tissue category, visceral category, central nervous system and skeletal spread.
- Time to axillary recurrence is the time between the date of randomisation and the date of axillary recurrence, measured in days.

For peer review only

- Axillary recurrence free survival is the time between the date of randomisation and date of confirmed axillary recurrence or date of death, whichever comes first, measured in days.
- Disease free survival; defined as the time between the date of randomisation and the date of disease progression (i.e. local or regional recurrence or distant metastasis) or death, whichever comes first, measured in days.
- Overall survival is the time between the date of randomisation and the date of death from any cause.
- Contralateral breast cancer is new primary malignancy in the opposite breast unless obviously contiguous with recurrent chest wall disease or proven on cytology/biopsy to be of metastatic origin.
- Non-breast cancer is any new non-breast primary malignancy, except for adequately treated, superficial squamous or basal cell carcinoma of the skin, or carcinoma in situ of the cervix.

Secondary outcomes are assessed at the following time points:

**Secondary Outcome**	**Assessment time point (months)**						
	**3**	**6**	**12**	**24**	**36**	**48**	**60**
Arm morbidity		X	X	X	X		
Quality of life	X	X	X	X	X		
Anxiety	X	X	X	X	X		
NHS costs	X	X	X	X	X	X	X
EQ-5D-5L	X	X	X	X	X		
Incremental cost per QALY gained					X		
Incremental cost per reduction in axillary recurrence							X
Local (breast or chest wall) recurrence		X	X	X	X	X	X
Regional (nodal) recurrence		X	X	X	X	X	X
Distant metastasis		X	X	X	X	X	X
Time to axillary recurrence		X	X	X	X	X	X
Axillary recurrence free survival		X	X	X	X	X	X
Disease free survival		X	X	X	X	X	X

For peer review only

Overall survival		X	X	X	X	X	X
Contralateral breast cancer		X	X	X	X	X	X
Non-breast malignancy		X	X	X	X	X	X

Participant Identification: Potential trial participants are identified at multi-disciplinary meetings. A participant information leaflet (PIL) and video is given to the participant

Based on local hospital practices, there are four participant pathways for recruitment:

(i) After primary breast surgery: Women who have a positive sentinel node are approached with regard to trial participation at the first post-operative clinic visit to offer trial participation.

(ii) Before primary breast surgery: In some hospitals sentinel node assessment is performed during primary breast surgery. In these hospitals, women are approached about the trial before surgery. If they are willing to participate consent is obtained prior to surgery. Randomisation is intra-operative if the SNs are confirmed as positive.

(iii) Post-chemotherapy: Women may be approached about the trial during and after chemotherapy but before starting axillary treatment (ANC or ART).

(iv) SNB prior to neoadjuvant therapy: Where the sentinel node biopsy is performed prior to neoadjuvant therapy patients can be approached during or after neoadjuvant therapy but prior to axillary treatment (ANC or ART).

Study Assessments

- **Baseline:** Baseline data are collected from the participant's medical notes and questionnaires.
- **Follow-up**: Follow-up assessments are at 6, 12, 24, 36, 48 and 60-months post randomisation with an additional postal questionnaire at 3-months.

The 6, 12, 24, 36, 48 and 60 month follow-up is in person or over the telephone, according to local practice. If contact cannot be made with the participant then medical notes can be used.

Data Collection: Trial data are entered at site into electronic case report forms (eCRF). Participants are identified only by their unique participant ID number. Access to the database is restricted and secure.

Data from routine medical notes can be used to collect outcomes for those who have been lost to follow-up or no longer wish to attend research visits.

Monitoring of Quality and Safety: Data handling and trial monitoring procedures are detailed in trial-specific data management and trial monitoring plans. Data quality and compliance with the protocol is assessed throughout the trial by central monitoring and site visits where required. The trial will undergo periodic audits by the trial's unit quality assurance team and the trial sponsor.

All reportable Serious Adverse Events are sent directly to the trial's unit and assessed by the Chief Investigator and reported as part of annual reports.

For peer review only

Confidentiality: Data obtained as a result of this study is considered confidential and confidentiality is ensured by utilising identification code numbers to correspond to treatment data.

Flagging with NHS Digital: Trial participation ends after 60 months with no further post-trial care (beyond standard practice). However, all women recruited to the study in the UK will be 'flagged' after discharge through the Data Linkage and Extract Service at NHS Digital to maximise outcome data collection and enable long-term follow-up data (greater than five years) to be reported.

Statistics

Sample Size: Among women who have adjuvant therapy plus axillary treatment, 2% have axillary recurrence within five years[11]. A total of 1700 women is required for analysis (850 in each group) in order to detect a non-inferiority margin of two percentage points for the risk difference using the Miettinen-Nurminen score interval[12], with 80% power and 2.5% one-sided alpha. Allowing for up to 10% non-compliance with treatment allocation and non-collection of primary outcome data, the target sample size to be randomised is 1900.

The magnitude of the non-inferiority margin was determined following consultation with relevant breast cancer consumer groups, lay people, breast surgeons and oncologists. There was consensus across all groups that an absolute increase in axillary recurrence of up to 2% is an acceptable trade-off for the increase in arm morbidity associated with axillary treatment.

Statistical Analysis: Analysis will be at the end of the five years follow-up of the last recruited participant. A detailed statistical analysis plan (SAP) will be developed by the trial statisticians in consultation with the Trial Management Group, and agreed with the Trial Steering Committee (TSC) before database lock and unblinding of data.

Descriptive statistics of demographic and clinical measures will be used to assess balance between the randomised arms at baseline. Continuous variables will be summarised in terms of the mean, standard deviation, median, lower and upper quartiles, minimum, maximum and number of observations. Categorical variables will be summarised in terms of frequency counts and percentages.

The primary approach for between-group comparative analyses of primary and secondary endpoints will be per protocol, and we will present point estimates and 95% confidence intervals. Sensitivity analysis where participants are analysed as randomised, regardless of compliance with allocation, will also be conducted for the primary outcome.

The primary analysis will assess whether adjuvant therapy alone is non-inferior to adjuvant therapy plus axillary treatment based on the primary endpoint of axillary recurrence within five years. The risk difference will be estimated using a mixed effects generalised linear model for binary outcome, with treatment arm and minimisation variables as fixed effects and recruitment site a random effect. Non-inferiority of adjuvant therapy alone compared with adjuvant therapy plus axillary treatment will be accepted if the upper boundary of the Miettinen-Nurminen two-sided 95% confidence limit for estimated difference in axillary recurrence is no greater than 2%.

We will conduct subgroup analyses of axillary recurrence and arm morbidity by fitting appropriate interaction terms in the regression models, according to the following variables

For peer review only

measured at baseline: number of sentinel node macrometastases; age; type of breast surgery; estrogen receptor (ER) status; tumour grade; sentinel node assessment technique; extranodal invasion. We will present subgroup-specific effects and 95% confidence intervals, as well as interactions, 95% confidence intervals and p-values. Due to the limited power available to detect all except large interactions, these subgroup analyses will be regarded as exploratory and interpreted with due caution

Analyses for surgical complications following treatment of ANC will be of a descriptive nature.

Economic Analysis: The economic evaluation will be carried out from the perspective of the NHS over a 3-year and 5-year time horizon. A 3-year time horizon was adopted for the cost-utility analysis as it was anticipated, that by 3-years post-randomisation, any differences in QoL between the intervention and standard care participants which might have been present earlier will have disappeared. Costs will be based upon the costs of the randomised interventions received and on the use of subsequent care and services.

For each participant, all clinical events (i) relevant to management and consequences following the initial sentinel node biopsy (adjuvant therapy alone or adjuvant therapy plus axillary treatment) (ii) up to the end of follow-up (iii) which occur in the hospital setting, will be recorded. These include primary treatments, re-admissions for complications or recurrences and associated adverse events. Events deemed unrelated to the consequences of the sentinel node biopsy or axillary treatment will not be included. In addition to these clinical events, participants will be asked to recall the extent to which they accessed primary care (e.g. GP) or other non-hospital services (e.g. physiotherapy visits) for their breast cancer at 6, 12, 24, 36, 48 and 60 months post-randomisation. Information relating to healthcare resource use will be collected via the eCRF. Costs will be estimated using routine sources (e.g. NHS reference costs, PSSRU). On the basis of each participant's record and the costs collected from routine sources, a total hospital cost and a total non-hospital cost will be derived for each participant for the duration of follow-up. These total costs will be combined to estimate the total cost per participant from which the total average cost per randomised group will be calculated. The breast and sentinel node biopsy procedure, systemic treatment, radiotherapy to breast or chest wall will not be included in the determination of costs, because all participants will undergo these treatment.

1) Cost-effectiveness analysis based on the incremental cost per reduction in axillary recurrence. Mean costs for each randomised arm will be calculated as will mean axillary recurrence and will be presented as point estimates of mean incremental costs and effects (reduced axillary recurrences) and the incremental cost per reduction in axillary recurrence at 5-years post-randomisation.

2) Cost-utility analysis based on the incremental cost per QALY gained. QALYs will be based on the area under the curve approach[13] using responses to the EQ-5D-5L completed at scheduled time points; baseline, 3, 6, 12, 24, and 36 months post-randomisation. The total average QALYs accruing in the intervention and standard care groups will be analysed for significant differences, following allowances for mortality (if occurring). Both mean cost and QALYs will be presented for each randomised group and incremental mean costs and QALY calculated along with the incremental cost per QALY gained at 3-years post-randomisation.

For peer review only

Both deterministic and stochastic sensitivity analyses will be used to address any uncertainty in our estimation of costs, effects and cost-effectiveness. Any uncertainty in the results, based on the stochastic sensitivity analyses, will be illustrated using cost-effectiveness planes and cost-effectiveness acceptability curves. Costs and effects will be discounted at UK recommended rates[14] as they are being estimated beyond a 1-year time horizon.

Radiotherapy Quality Assurance (RT QA): The UK Radiotherapy Trials QA (RTTQA) Group will be responsible for implementing and coordinating the RT QA programme through a named contact for the trial. The programme includes pre-trial and on-trial activities and is outlined below:

Pre-trial QA - Prior to site activation:

- Facility questionnaire – Details of treatment technique, immobilisation, verification and dosimetry will be recorded.

- Dummy Run - All centres must submit a test participant case to the RTTQA group, for review, demonstrating planned radiotherapy treatment to the breast/chest wall, axilla and supraclavicular fossa. The outlining, planning and treatment of this participant case should reflect how the centre intends to plan and treat all participants recruited to POSNOC.

On-trial QA – Ongoing QA during trial recruitment:

- Retrospective individual case reviews – All centres must export radiotherapy plan data to the RTTQA Group for their first ten participants. Data will include participant history, CT data, structure set, plan and dose files. The retrospective review process should include at least three plans with radiotherapy to the nodal region.

- Ongoing data collection - Radiotherapy data related to the RT QA programme will be collected and stored by the RTTQA Group for all participants, regardless of study allocation. A random sample of plans will be reviewed from each site during the trial recruitment period.

The sites will be required to adhere to the POSNOC radiotherapy planning and delivery guidelines when treating participants in the study. The RTTQA Group will promote and monitor compliance to the guidelines for the duration of trial recruitment by systematically reviewing a sample of radiotherapy plans submitted by centres. Radiotherapy guideline deviations, non-compliance or areas of concern will be recorded and if required, discussed with the TMG and individual centres.

Patient and public involvement: As part of the development of the project and protocol, women from the Derby Breast Cancer Support Group, Bosom Buddies (Guildford Breast Cancer Support Group) and Independent Cancer Patients' Voice (ICPV) have commented at all stages on the design and acceptability of the study. The Breast Cancer Care and Lymphoedema Support Network have had the opportunity to contribute to this protocol.

Independent Cancer Patients' Voice is an independent patient advocate group (http://independentcancerpatientsvoice.org.uk/). Two members of ICPV will provide a patient perspective throughout the duration of the study.

For peer review only

Dissemination plan

The dissemination of the study results will be via a study report and research papers for publication in peer reviewed journals, and presentation at relevant conferences. Reporting will be in compliance with CONSORT recommendations. Publication of the results will be based on outcomes at 5-years following the last recruited participant.

A summary of the results will be made available to participants through a newsletter (unless they state they do not wish to receive this), and will also be publicised through Independent Cancer Patients' Voice, Cancer Research UK, Breast Cancer Now and Lymphoedema Support Network.

Trial status: Recruiting. On 09 June 2021, 1866 patients have been included.

Data sharing plan: Requests for data collected for POSNOC will be considered by the TMG and NCTU Data Sharing review panel.

Ethics approval: The study has been approved by the National Research Ethics Service (NRES) Committee East Midlands – Nottingham 2 (REC reference: 13/EM/0459)

Additional Documentation: The current full trial protocol (v 6.0 26 March 2020), details of previous protocol amendments, copies of current PIL and informed consent forms, oversight committee charters and a full list of participating sites can be found at www.posnoc.co.uk.

Author contributions

Contributors: AG conceived the study and prepared the first draft of the protocol. All authors contributed to the study design. All authors read, critically revised and approved the manuscript before submission.

Footnotes

Trial Management Group members: Amit Goyal (Chair), Shabina Sadiq, Mickey Lewis, Rachel Haines, Alan Montgomery, Wei Tan, G Bruce Mann, Kathryn Monson, Valerie Jenkins, Patricia Fairbrother, David Dodwell, Tara Homer, Heath Badger, Zohal Nabi, Romaana Mir, Elizabeth Miles, Luke Vale, Malcolm W Reed, Lesley Fallowfield, Robert Coleman, Vaughan Keeley, Ian Ellis, Daniel Davis, Teresa Grieve (past members: Lelia Duley, Diane Whitham, Margaret Childs, Apostolos Fakis, Robert G Newcombe, David Whynes, Eleanor Mitchell, Rebecca Haydock, Clare Brittain, Clare Upton, Sarah Craig)

Nottingham Clinical Trials Unit staff: Alan Montgomery, Rachel Haines, Shabina Sadiq, Wei Tan, Mickey Lewis, Aisha Shafayat, Charlotte Gidman, Mara Ozolins, Lelia Duley, Diane Whitham, Margaret Childs, Eleanor Mitchell, Rebecca Haydock, Clare Brittain, Clare Upton, Sarah Craig

Patient Reported Outcomes staff (Sussex Health Outcomes Research & Education in Cancer (SHORE-C)): Kathryn Monson, Valerie Jenkins, Lesley Fallowfield

Health Economics staff (Newcastle University): Tara Homer, Luke Vale (past staff: Laura Ternent)

For peer review only

Breast Cancer Trials Australia and New Zealand staff: Heath Badger, Bruce Mann, Nicole Francis, Flonda Probert, Lisa Paksec, Rose Lucas (past staff: Annette Dempsey, Rochelle Thornton)

Radiotherapy Quality Assurance team: Daniel Megias, Zohal Nabi, Preetinder Singh, Andrei Caraman, Elizabeth Miles, Romaana Mir, Roeum Butt, Hannah Price

Independent Trial Steering Committee members: Alastair Thompson (Chair), Alistair Ring, Nisha Sharma, Peter Barrett Lee, Julie Wolfarth, Ian White, Ramsey Cutress (past members:

Independent Data Monitoring Committee members: Judith Bliss (chair), Daniel Rea, Peter Dubsky

Sponsor: The trial is sponsored by the University Hospitals of Derby and Burton NHS Foundation Trust (uhdb.sponsor@nhs.net**)**. Sponsor staff - Daniel Davis, Joanne Thornhill, Anne Shaw, Amy Farmer, Teresa Grieve

Acknowledgments: We thank the patients who are participating in this trial, and staff at the participating centres and the independent data monitoring committee and trial steering committee for overseeing the trial.

Funding: This project is funded by the National Institute for Health Research Health Technology Assessment Programme (project number 12/35/17). Australian and New Zealand participation is funded by National Health and Medical Research Council (NHMRC) (project grant 1083172).

Disclaimer: The views expressed are those of the authors and not necessarily those of the Health Technology Assessment Programme, NIHR, NHS or the Department of Health and Social Care.

Competing interests: LV was a member of the NIHR Health Technology Assessment Programme, Clinical Trials and Evaluation Panel from 2014-2018.

Reference List

1. Buchholz, T.A., et al., *Impact of systemic treatment on local control for patients with lymph node-negative breast cancer treated with breast-conservation therapy.* J.Clin.Oncol., 2001. **19**(8): p. 2240-2246.
2. Mansel, R.E., et al., *Results of a national training programme in sentinel lymph node biopsy for breast cancer.* Br J Surg, 2013. **100**(5): p. 654-61.
3. Khonji, N.I., et al., *Incidence of nodes in completion mastectomy specimens following breast conservation and axillary clearance.* Br.J.Surg., 2002. **89**(10): p. 1294-1295.
4. Giuliano, A.E., et al., *Locoregional recurrence after sentinel lymph node dissection with or without axillary dissection in patients with sentinel lymph node metastases: the American College of Surgeons Oncology Group Z0011 randomized trial.* Ann Surg, 2010. **252**(3): p. 426-32; discussion 432-3.
5. Azghadi, S., M. Daly, and J. Mayadev, *Practice Patterns of Radiation Field Design for Sentinel Lymph Node-Positive Early-Stage Breast Cancer.* Clin.Breast Cancer, 2016. **16**(5): p. 410-417.
6. Donker, M., et al., *Radiotherapy or surgery of the axilla after a positive sentinel node in breast cancer (EORTC 10981-22023 AMAROS): a randomised, multicentre, open-label, phase 3 non-inferiority trial.* Lancet Oncol, 2014. **15**(12): p. 1303-1310.

For peer review only

7. Armer, J.M., et al., *Predicting breast cancer-related lymphedema using self-reported symptoms.* Nurs.Res., 2003. **52**(6): p. 370-379.
8. Kennedy, C.A., et al., *Measurement properties of the QuickDASH (Disabilities of the Arm, Shoulder and Hand) outcome measure and cross-cultural adaptations of the QuickDASH: a systematic review.* Qual.Life Res., 2013.
9. Coster, S., K. Poole, and L.J. Fallowfield, *The validation of a quality of life scale to assess the impact of arm morbidity in breast cancer patients post-operatively.* Breast Cancer Res.Treat., 2001. **68**(3): p. 273-282.
10. Spielberger, C., *The Manual for the State-Trait Inventory.* Palo Alto, CA: Consulting Psychologists Press Inc., 1983.
11. Sanghani, M., E.M. Balk, and B. Cady, *Impact of axillary lymph node dissection on breast cancer outcome in clinically node negative patients: a systematic review and meta-analysis.* Cancer, 2009. **115**(8): p. 1613-20.
12. Miettinen, O. and M. Nurminen, *Comparative analysis of two rates.* Stat Med, 1985. **4**(2): p. 213-26.
13. Matthews, J.N., et al., *Analysis of serial measurements in medical research.* BMJ, 1990. **300**(6719): p. 230-235.
14. *National Institute for Health and Care Excellence (NICE): Guide to the methods of technology appraisal 2013.* https://www.nice.org.uk/article/pmg9, 2013.

For peer review only

POSNOC protocol BMJ open v 2.0

Figure 1. Trial flow chart

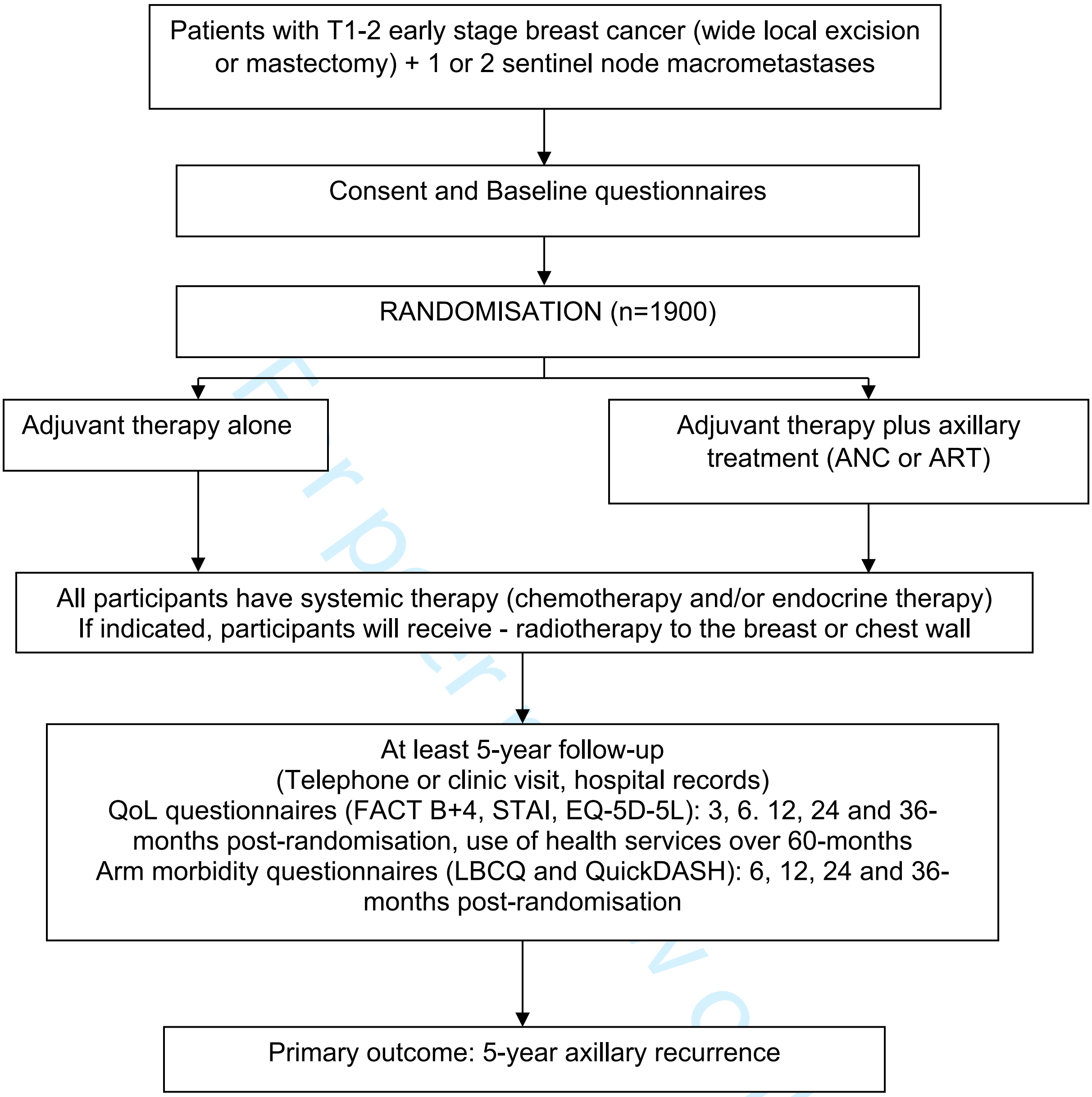

ANC, axillary node clearance; ART axillary radiotherapy; FACT B+4, Functional Assessment of Cancer Therapy – Breast +4; STAI, State-Trait Anxiety Inventory; LBCQ, Lymphoedema and Breast Cancer Questionnaire; QuickDASH, Disabilities of the Arm, Shoulder and Hand questionnaire.

BMJ Open

Protocol: POSNOC - Positive Sentinel Node: adjuvant therapy alone versus adjuvant therapy plus Clearance or axillary radiotherapy. A randomised controlled trial of axillary treatment in women with early stage breast cancer who have metastases in one or two sentinel nodes

Journal:	*BMJ Open*
Manuscript ID	bmjopen-2021-054365.R1
Article Type:	Protocol
Date Submitted by the Author:	24-Sep-2021
Complete List of Authors:	Goyal, Amit; Royal Derby Hospital,<br>Mann, G. Bruce; The University of Melbourne<br>Fallowfield, Lesley; University of Sussex, SHORE-C, Brighton & Sussex Medical School<br>Duley, Lelia; Nottingham Clinical Trials Unit, University of Nottingham<br>Reed, Malcolm; Brighton and Sussex Medical School,<br>Dodwell, David; Oxford University, Nuffield Department of Population Health<br>Coleman, Robert E.; Weston Park Hospital<br>Fakis, Apostolos; Royal Derby Hospital, Research and Development<br>Newcombe, Robert; Cardiff University, Department of Primary Care and Public Health<br>Jenkins, Valerie; University of Sussex,<br>Whitham, Diane; The University of Nottingham, Clinical Trials Unit<br>Childs, Margo; University of Nottingham,<br>Whynes, David; University of Nottingham<br>Keeley, Vaughn; University of Nottingham,<br>Ellis, Ian ; University of Nottingham, Division of Oncology, School of Medicine<br>Fairbrother, Patricia; Independent Cancer Patients' Voice,<br>Sadiq, Shabina; Nottingham Clinical Trials Unit, University of Nottingham<br>Monson, Kathryn; Brighton & Sussex Medical School, Sussex Health Outcomes, Research & Education in Cancer (SHORE-C)<br>Montgomery, Alan; Nottingham Clinical Trials Unit, University of Nottingham<br>Tan, Wei; University of Nottingham School of Medecine, Nottingham Clinical Trials Unit<br>Vale, Luke; Newcastle University, Health Economics Group, Population Health Sciences Institute<br>Homer, Tara; Newcastle University, Health Economics Group, Population Health Sciences Institute<br>Badger, Heath; Breast Cancer Trials<br>Haines, Rachel; University of Nottingham School of Medecine, Nottingham Clinical Trials Unit<br>Lewis, Mickey; Nottingham Clinical Trials Unit, University of Nottingham<br>Megias, Daniel; National Radiotherapy Trials Quality Assurance Group, Mount Vernon Cancer Centre

	Nabi, Zohal; National Radiotherapy Trials Quality Assurance Group, Mount Vernon Cancer Centre<br>Singh, Preetinder; National Radiotherapy Trials Quality Assurance Group, Mount Vernon Cancer Centre<br>Caraman, Andrei; National Radiotherapy Trials Quality Assurance Group, Mount Vernon Cancer Centre<br>Miles, Elizabeth; National Radiotherapy Trials Quality Assurance Group, Mount Vernon Hospital
**Primary Subject Heading**:	Surgery
Secondary Subject Heading:	Surgery
Keywords:	Breast surgery < SURGERY, Breast tumours < ONCOLOGY, Adult radiotherapy < RADIOTHERAPY

SCHOLARONE™ Manuscripts

I, the Submitting Author has the right to grant and does grant on behalf of all authors of the Work (as defined in the below author licence), an exclusive licence and/or a non-exclusive licence for contributions from authors who are: i) UK Crown employees; ii) where BMJ has agreed a CC-BY licence shall apply, and/or iii) in accordance with the terms applicable for US Federal Government officers or employees acting as part of their official duties; on a worldwide, perpetual, irrevocable, royalty-free basis to BMJ Publishing Group Ltd ("BMJ") its licensees and where the relevant Journal is co-owned by BMJ to the co-owners of the Journal, to publish the Work in this journal and any other BMJ products and to exploit all rights, as set out in our licence.

The Submitting Author accepts and understands that any supply made under these terms is made by BMJ to the Submitting Author unless you are acting as an employee on behalf of your employer or a postgraduate student of an affiliated institution which is paying any applicable article publishing charge ("APC") for Open Access articles. Where the Submitting Author wishes to make the Work available on an Open Access basis (and intends to pay the relevant APC), the terms of reuse of such Open Access shall be governed by a Creative Commons licence – details of these licences and which Creative Commons licence will apply to this Work are set out in our licence referred to above.

Other than as permitted in any relevant BMJ Author's Self Archiving Policies, I confirm this Work has not been accepted for publication elsewhere, is not being considered for publication elsewhere and does not duplicate material already published. I confirm all authors consent to publication of this Work and authorise the granting of this licence.

For peer review only

Title: Protocol: POSNOC - POsitive Sentinel NOde: adjuvant therapy alone versus adjuvant therapy plus Clearance or axillary radiotherapy. A randomised controlled trial of axillary treatment in women with early stage breast cancer who have metastases in one or two sentinel nodes

Authors: Amit Goyal[1], G Bruce Mann[2], Lesley Fallowfield[3], Lelia Duley[4], Malcolm W Reed[5], David Dodwell[6], Robert Coleman[7], Apostolos Fakis[1], Robert G Newcombe[8], Valerie Jenkins[3], Diane Whitham[4], Margo Childs[4], David Whynes[4], Vaughan Keeley[1], Ian Ellis[9], Patricia Fairbrother[10], Shabina Sadiq[4], Kathryn Monson[3], Alan A Montgomery[4], Wei Tan[4], Luke Vale[11], Tara Homer[11], Heath Badger[12], Rachel Haines[4], Mickey Lewis[4], Daniel Megias[13], Zohal Nabi[13], Preetinder Singh[13], Andrei Caraman[13], Elizabeth Miles[13] on behalf of the POSNOC Trial Management Group

Institutions: [1]University Hospitals of Derby and Burton NHS Foundation Trust, Derby, UK; [2]Victorian Comprehensive Cancer Centre, Melbourne, Australia; [3]SHORE-C, University of Sussex, Brighton, UK; [4]Nottingham Clinical Trials Unit, University of Nottingham, Nottingham, UK; [5]Brighton and Sussex Medical School, University of Sussex, Brighton, UK; [6]Nuffield Department of Population Health, University of Oxford, Oxford, UK; [7]Weston Park Hospital, Sheffield, UK; [8]Cardiff University, Cardiff, UK; [9]University of Nottingham, Nottingham, UK; [10]Independent Cancer Patients' Voice, London, UK; [11]Newcastle University, Newcastle, UK; [12]Breast Cancer Trials, Newcastle, Australia; [13]National Radiotherapy Trials QA (RTTQA) Group, Mount Vernon Cancer Centre, Northwood, UK

Correspondence: Amit Goyal (Chief Investigator), University Hospitals of Derby and Burton NHS Foundation Trust, Derby, UK. Email: amit.goyal@nhs.net

Tel: +44(0)1332 785538, Twitter handle: @_amit_goyal

Word count: 4000

Figures: 1

Tables: 1

For peer review only

For peer review only - http://bmjopen.bmj.com/site/about/guidelines.xhtml

Abstract

Introduction

ACOSOG-Z0011(Z11) trial showed that axillary node clearance(ANC) may be omitted in women with ≤2 positive nodes undergoing breast conserving surgery (BCS) and whole breast radiotherapy (RT). A confirmatory study is needed to clarify the role of axillary treatment in women with ≤2 macrometastases undergoing BCS and groups that were not included in Z11 e.g. mastectomy and those with microscopic extranodal invasion.

The primary objective of POSNOC is to evaluate whether for women with breast cancer and 1 or 2 macrometastases, adjuvant therapy alone is non-inferior to adjuvant therapy plus axillary treatment, in terms of 5-year axillary recurrence.

Methods and analysis

POSNOC is a pragmatic, multicentre, non-inferiority, international trial with participants randomised in a 1:1 ratio. Women are eligible if they have T1/T2, unifocal or multifocal invasive breast cancer, and 1 or 2 macrometastases at sentinel node biopsy, with or without extranodal extension. In the intervention group women receive adjuvant therapy alone, in the standard care group they receive ANC or axillary RT. In both groups women receive adjuvant therapy, according to local guidelines. This includes systemic therapy and, if indicated, RT to breast or chest wall. The UK Radiotherapy Trials Quality Assurance group manages the in-built Radiotherapy Quality Assurance programme. Primary endpoint is 5-year axillary recurrence. Secondary outcomes are arm morbidity assessed by LBCQ and QuickDASH questionnaires; Quality of Life and anxiety as assessed with FACT B+4 and STAI questionnaires respectively; other oncological outcomes; economic evaluation using EQ-5D-5L. Target sample size is 1900. Primary analysis is per protocol. Recruitment started on 1 August 2014 and as of 09 June 2021, 1866 participants have been randomised.

Ethics and dissemination: Protocol was approved by the National Research Ethics Service Committee East Midlands – Nottingham 2 (REC reference: 13/EM/0459). Results will be submitted for publication in peer-reviewed journals.

Trial registration number: ISRCTN54765244; NCT02401685

Strengths and limitations of this study

- POSNOC includes women undergoing mastectomy and those with extranodal invasion
- Only women with 1 or 2 macrometastases are eligible for randomisation

For peer review only - http://bmjopen.bmj.com/site/about/guidelines.xhtml

- POSNOC has an in-built radiotherapy quality assurance programme co-ordinated by the UK Radiotherapy Trials QA (RTTQA) Group
- POSNOC is an international trial - UK, Australia and New Zealand

Key words: POSNOC, sentinel node biopsy, breast cancer, axillary node clearance, axillary radiotherapy

For peer review only

For peer review only - http://bmjopen.bmj.com/site/about/guidelines.xhtml

Introduction

Currently, women with early breast cancer and 1 or 2 macrometastases in sentinel lymph nodes (SNs) generally undergo some form of axillary treatment, either surgical axillary node clearance (ANC) or radiotherapy to the axilla (ART). The use of axillary treatment is based on the assumption that it reduces the risk of axillary recurrence and might improve survival. However, axillary treatment damages lymphatic drainage from the arm and women can subsequently develop lymphoedema, restricted shoulder movement, pain, numbness, and other sensory problems. These adverse effects interfere with daily activities, are distressing, impair quality of life (QoL) and are costly to the NHS in terms of rehabilitative treatments, as they are often permanent and symptom relief is difficult.

Axillary treatment may no longer be necessary for this group of women, as they usually have a low axillary tumour burden and current systemic therapy is effective at preventing breast and axillary recurrence[1]. Also, sentinel node biopsy has already removed the lymph nodes most likely to have metastasis[2]. Moreover, when adjuvant therapy includes radiotherapy to the breast or chest wall, the lower axilla may be treated as it is often included in the tangential irradiation field, and some lower level axillary nodes may be removed at mastectomy[3].

This hypothesis is supported by the Z11 trial[4] that showed that ANC may be omitted in women with ≤2 positive nodes undergoing breast conserving surgery (BCS) and receiving whole breast RT. Axillary recurrence was low, and there were no clear differences between the two groups (ANC 0.5% vs. no ANC 0.9%) at 6.3 years. Z11 has not changed clinical practice because of several limitations. The study did not meet its recruitment goal, approximately 40% of participants had micrometastases, many were lost to follow-up (19.4%), and there was a lack of radiation therapy quality assurance. Most US radiation oncologists continue to treat the undissected axilla in women with micro or macro metastases with ART[5]. Therefore, it appears that clinicians in the US are interpreting Z11 in line with AMAROS study which showed that ART is a less morbid alterative to axillary lymph node dissection (ALND) in women with low volume nodal disease[6] rather than omitting axillary treatment. Furthermore, the results are not applicable to subgroups that were not included such as those with mastectomy and microscopic extranodal invasion.

For peer review only - http://bmjopen.bmj.com/site/about/guidelines.xhtml

Objective

The primary objective of the POSNOC trial is to evaluate whether, for women with early breast cancer and one or two nodes with macrometastases, adjuvant therapy alone is non-inferior to adjuvant therapy plus axillary treatment (ANC or ART), in terms of 5-year axillary recurrence.

Methods and analysis

Design: POSNOC is a pragmatic randomised, multicentre, non-inferiority, international (UK, Australia, New Zealand) trial. Target sample size is 1900 participants, with 5-year follow-up. Flow of participants through the trial is summarised in Figure 1.

Randomisation: Participants are allocated via a secure internet-based system, using minimisation that is weighted toward minimising imbalance between trial arms with probability 0.8. Allocation is stratified by recruiting site and minimised by the following variables: age (<50, ≥50 years); type of breast surgery (breast-conserving surgery (BCS), mastectomy); estrogen receptor status (ER) (positive, negative); number of positive nodes (1, 2); intra-operative sentinel assessment using OSNA (yes, no).

Target population: Women with unifocal or multifocal invasive breast cancer with the largest lesion ≤5cm, who have 1 or 2 sentinel nodes with macrometastases (>2mm), with or without extranodal invasion.

Women are usually screened for eligibility after their initial breast surgery and sentinel node biopsy. In hospitals where sentinel node histology is assessed intra-operatively, women are asked to give their consent pre-operatively and then randomised intra-operatively if their sentinel nodes are found to be positive.

Inclusion Criteria: Women are eligible for inclusion if ALL of the following criteria apply:

- 18 years or older
- Unifocal or multi-focal invasive tumour ≤5 cm in its largest dimension, measured pathologically or largest invasive tumour diameter on radiology for women who are randomised intra-operatively or who undergo sentinel node biopsy before neoadjuvant therapy (tumour size is based only on the single largest tumour)
- At sentinel node biopsy have 1 or 2 nodes with macrometastases (tumour deposit >2.0mm in largest dimension or defined as macrometastases on molecular assay)
- Fit for axillary treatment and adjuvant therapy
- Have given informed consent

Exclusion Criteria: Women are excluded if they have any of the following:

For peer review only - http://bmjopen.bmj.com/site/about/guidelines.xhtml

- Bilateral invasive breast cancer
- more than 2 nodes with macrometastases
- neoadjuvant therapy for breast cancer except
  - o if sentinel node biopsy performed prior to neoadjuvant therapy in women with early breast cancer
  - o short duration of neoadjuvant endocrine therapy (up to 3-months)
- previous axillary surgery on the same body side as the scheduled sentinel node biopsy
- not planned to receive adjuvant systemic therapy
- previous cancer less than 5-years previously or concomitant malignancy except
  - o basal or squamous cell carcinoma of the skin
  - o in situ carcinoma of the cervix
  - o in situ melanoma
  - o contra- or ipsilateral in situ breast cancer

Trial treatment arms: All participants receive adjuvant therapy according to local guidelines. This includes chemotherapy and/or endocrine therapy for all women, and radiotherapy to breast or chest wall if indicated. Human epidermal growth factor receptor 2 (HER2) targeted treatment is administered when indicated.

Trial participants are allocated to one of the following two groups:

(i) Adjuvant therapy alone (intervention group)

See above for adjuvant therapy. Axillary and supraclavicular fossa radiotherapy is not allowed.

(ii) Adjuvant therapy plus axillary treatment (standard care group)

See above for adjuvant therapy. Axillary treatment can be axillary node clearance or axillary radiotherapy as per local guidelines.

Outcomes

Primary Outcome: The primary outcome is 5-year axillary recurrence defined as pathologically (cytology or biopsy) and/or radiologically confirmed recurrence in lymph nodes draining the primary tumour site.

Secondary Outcomes (Table 1)**:**

- Arm morbidity assessed by a) Lymphoedema and Breast Cancer Questionnaire (LBCQ)[7] - the definition of lymphoedema is 'yes' to the two questions -'heaviness during the past year' and 'swelling now'; b) QuickDASH questionnaire[8] - Shoulder, arm and hand disability is defined as a change from baseline in the QuickDASH score of at least 14 points.

For peer review only - http://bmjopen.bmj.com/site/about/guidelines.xhtml

- QoL assessed by Functional Assessment of Cancer Therapy-Breast+4 (FACT B+4) questionnaire[9]. The total FACT B+4 score reflects global QoL and comprises physical (7 items), functional (7 items), social (7 items) and emotional well-being (6 items) plus the breast cancer concerns and arm morbidity scale (14 items) (total of 41 items; total score ranges from 0-164). The 5 item arm morbidity subscale score comprises the sum of the scores from items B3, B10, B11, B12, B13 (range 0-20) and will be analysed separately. Analyses will include the proportion of participants in the two allocated treatment groups reporting 'somewhat', 'quite a bit' and 'very much' for each of the 5 items.
- Anxiety assessed by using the Spielberger State/Trait Anxiety Inventory(STAI)[10]. The STAI consists of 2 questionnaires with 20 items. It assesses anxiety proneness (Trait) and the current state of anxiety or anxiety change (State). Each item is rated on a four point Likert scale. High STAI scores signify greater anxiety. The Trait anxiety is measured only once and the State at each time point.
- Economic outcomes will include costs to the NHS, quality-adjusted life years (QALYs) based on responses to the EQ-5D-5L, and the primary outcome, axillary recurrence. For more details, see Economic Analysis section below.
- Local (breast or chest wall) recurrence; defined as pathologically (cytology or biopsy) and/or radiologically confirmed recurrence after mastectomy in the skin or soft tissue of the chest wall within the anatomical area bounded by the mid-sternal line, the clavicle, the posterior axillary line and the costal margin or any type of breast carcinoma in the breast after conservation therapy.
- Regional (nodal) recurrence; defined as pathologically (cytology or biopsy) and/or radiologically confirmed recurrent tumour in the lymph nodes in the ipsilateral axilla, infraclavicular, supraclavicular fossa, interpectoral area or ipsilateral internal mammary chain.
- Distant metastasis; defined as confirmed metastasis (positive pathology and/or definitive evidence on imaging) in all other sites of recurrence and may include those classified as: soft-tissue category, visceral category, central nervous system and skeletal spread.
- Time to axillary recurrence is the time between the date of randomisation and the date of axillary recurrence, measured in days.

For peer review only - http://bmjopen.bmj.com/site/about/guidelines.xhtml

For peer review only

- Axillary recurrence free survival is the time between the date of randomisation and date of confirmed axillary recurrence or date of death, whichever comes first, measured in days.
- Disease free survival; defined as the time between the date of randomisation and the date of disease progression (i.e. local or regional recurrence or distant metastasis) or death, whichever comes first, measured in days.
- Overall survival is the time between the date of randomisation and the date of death from any cause.
- Contralateral breast cancer is new primary malignancy in the opposite breast unless obviously contiguous with recurrent chest wall disease or proven on cytology/biopsy to be of metastatic origin.
- Non-breast cancer is any new non-breast primary malignancy, except for adequately treated, superficial squamous or basal cell carcinoma of the skin, or carcinoma in situ of the cervix.

Table 1. Secondary outcomes are assessed at the following time points:

Secondary Outcome	Assessment time point (months)						
	3	6	12	24	36	48	60
Arm morbidity		X	X	X	X		
Quality of life	X	X	X	X	X		
Anxiety	X	X	X	X	X		
NHS costs	X	X	X	X	X	X	X
EQ-5D-5L	X	X	X	X	X		
Incremental cost per QALY gained					X		
Incremental cost per reduction in axillary recurrence							X
Local (breast or chest wall) recurrence		X	X	X	X	X	X
Regional (nodal) recurrence		X	X	X	X	X	X
Distant metastasis		X	X	X	X	X	X
Time to axillary recurrence		X	X	X	X	X	X
Axillary recurrence free survival		X	X	X	X	X	X
Disease free survival		X	X	X	X	X	X

For peer review only - http://bmjopen.bmj.com/site/about/guidelines.xhtml

Overall survival		X	X	X	X	X	X
Contralateral breast cancer		X	X	X	X	X	X
Non-breast malignancy		X	X	X	X	X	X

Participant Identification: Potential trial participants are identified at multi-disciplinary meetings. A participant information leaflet (PIL) and video is given to the participant

Based on local hospital practices, there are four participant pathways for recruitment:

(i) After primary breast surgery: Women who have a positive sentinel node are approached with regard to trial participation at the first post-operative clinic visit to offer trial participation.

(ii) Before primary breast surgery: In some hospitals sentinel node assessment is performed during primary breast surgery. In these hospitals, women are approached about the trial before surgery. If they are willing to participate consent is obtained prior to surgery. Randomisation is intra-operative if the SNs are confirmed as positive.

(iii) Post-chemotherapy: Women may be approached about the trial during and after chemotherapy but before starting axillary treatment (ANC or ART).

(iv) SNB prior to neoadjuvant therapy: Where the sentinel node biopsy is performed prior to neoadjuvant therapy patients can be approached during or after neoadjuvant therapy but prior to axillary treatment (ANC or ART).

Study Assessments

- **Baseline:** Baseline data are collected from the participant's medical notes and questionnaires.
- **Follow-up**: Follow-up assessments are at 6, 12, 24, 36, 48 and 60-months post randomisation with an additional postal questionnaire at 3-months.

The 6, 12, 24, 36, 48 and 60 month follow-up is in person or over the telephone, according to local practice. If contact cannot be made with the participant then medical notes can be used.

Data Collection: Trial data are entered at site into electronic case report forms (eCRF). Participants are identified only by their unique participant ID number. Access to the database is restricted and secure.

Data from routine medical notes can be used to collect outcomes for those who have been lost to follow-up or no longer wish to attend research visits.

Monitoring of Quality and Safety: Data handling and trial monitoring procedures are detailed in trial-specific data management and trial monitoring plans. Data quality and compliance with the protocol is assessed throughout the trial by central monitoring and site visits where required. The trial will undergo periodic audits by the trial's unit quality assurance team and the trial sponsor.

All reportable Serious Adverse Events are sent directly to the trial's unit and assessed by the Chief Investigator and reported as part of annual reports.

For peer review only - http://bmjopen.bmj.com/site/about/guidelines.xhtml

Confidentiality: Data obtained as a result of this study is considered confidential and confidentiality is ensured by utilising identification code numbers to correspond to treatment data.

Flagging with NHS Digital: Trial participation ends after 60 months with no further post-trial care (beyond standard practice). However, all women recruited to the study in the UK will be 'flagged' after discharge through the Data Linkage and Extract Service at NHS Digital to maximise outcome data collection and enable long-term follow-up data (greater than five years) to be reported.

Statistics

Sample Size: Among women who have adjuvant therapy plus axillary treatment, 2% have axillary recurrence within five years[11]. A total of 1700 women is required for analysis (850 in each group) in order to detect a non-inferiority margin of two percentage points for the risk difference using the Miettinen-Nurminen score interval[12], with 80% power and 2.5% one-sided alpha. Allowing for up to 10% non-compliance with treatment allocation and non-collection of primary outcome data, the target sample size to be randomised is 1900.

The magnitude of the non-inferiority margin was determined following consultation with relevant breast cancer consumer groups, lay people, breast surgeons and oncologists. There was consensus across all groups that an absolute increase in axillary recurrence of up to 2% is an acceptable trade-off for the increase in arm morbidity associated with axillary treatment.

Statistical Analysis: Analysis will be at the end of the five years follow-up of the last recruited participant. A detailed statistical analysis plan (SAP) will be developed by the trial statisticians in consultation with the Trial Management Group, and agreed with the Trial Steering Committee (TSC) before database lock and unblinding of data.

Descriptive statistics of demographic and clinical measures will be used to assess balance between the randomised arms at baseline. Continuous variables will be summarised in terms of the mean, standard deviation, median, lower and upper quartiles, minimum, maximum and number of observations. Categorical variables will be summarised in terms of frequency counts and percentages.

The primary approach for between-group comparative analyses of primary and secondary endpoints will be per protocol, and we will present point estimates and 95% confidence intervals. Sensitivity analysis where participants are analysed as randomised, regardless of compliance with allocation, will also be conducted for the primary outcome.

The primary analysis will assess whether adjuvant therapy alone is non-inferior to adjuvant therapy plus axillary treatment based on the primary endpoint of axillary recurrence within five years. The risk difference will be estimated using a mixed effects generalised linear model for binary outcome, with treatment arm and minimisation variables as fixed effects and recruitment site a random effect. Non-inferiority of adjuvant therapy alone compared with adjuvant therapy plus axillary treatment will be accepted if the upper boundary of the Miettinen-Nurminen two-sided 95% confidence limit for estimated difference in axillary recurrence is no greater than 2%.

We will conduct subgroup analyses of axillary recurrence and arm morbidity by fitting appropriate interaction terms in the regression models, according to the following variables

For peer review only - http://bmjopen.bmj.com/site/about/guidelines.xhtml

For peer review only

measured at baseline: number of sentinel node macrometastases; age; type of breast surgery; estrogen receptor (ER) status; tumour grade; sentinel node assessment technique; extranodal invasion. We will present subgroup-specific effects and 95% confidence intervals, as well as interactions, 95% confidence intervals and p-values. Due to the limited power available to detect all except large interactions, these subgroup analyses will be regarded as exploratory and interpreted with due caution

Analyses for surgical complications following treatment of ANC will be of a descriptive nature.

Economic Analysis: The economic evaluation will be carried out from the perspective of the NHS over a 3-year and 5-year time horizon. A 3-year time horizon was adopted for the cost-utility analysis as it was anticipated, that by 3-years post-randomisation, any differences in QoL between the intervention and standard care participants which might have been present earlier will have disappeared. Costs will be based upon the costs of the randomised interventions received and on the use of subsequent care and services.

For each participant, all clinical events (i) relevant to management and consequences following the initial sentinel node biopsy (adjuvant therapy alone or adjuvant therapy plus axillary treatment) (ii) up to the end of follow-up (iii) which occur in the hospital setting, will be recorded. These include primary treatments, re-admissions for complications or recurrences and associated adverse events. Events deemed unrelated to the consequences of the sentinel node biopsy or axillary treatment will not be included. In addition to these clinical events, participants will be asked to recall the extent to which they accessed primary care (e.g. GP) or other non-hospital services (e.g. physiotherapy visits) for their breast cancer at 6, 12, 24, 36, 48 and 60 months post-randomisation. Information relating to healthcare resource use will be collected via the eCRF. Costs will be estimated using routine sources (e.g. NHS reference costs, PSSRU). On the basis of each participant's record and the costs collected from routine sources, a total hospital cost and a total non-hospital cost will be derived for each participant for the duration of follow-up. These total costs will be combined to estimate the total cost per participant from which the total average cost per randomised group will be calculated. The breast and sentinel node biopsy procedure, systemic treatment, radiotherapy to breast or chest wall will not be included in the determination of costs, because all participants will undergo these treatment.

1) Cost-effectiveness analysis based on the incremental cost per reduction in axillary recurrence. Mean costs for each randomised arm will be calculated as will mean axillary recurrence and will be presented as point estimates of mean incremental costs and effects (reduced axillary recurrences) and the incremental cost per reduction in axillary recurrence at 5-years post-randomisation.

2) Cost-utility analysis based on the incremental cost per QALY gained. QALYs will be based on the area under the curve approach[13] using responses to the EQ-5D-5L completed at scheduled time points; baseline, 3, 6, 12, 24, and 36 months post-randomisation. The total average QALYs accruing in the intervention and standard care groups will be analysed for significant differences, following allowances for mortality (if occurring). Both mean cost and QALYs will be presented for each randomised group and incremental mean costs and QALY calculated along with the incremental cost per QALY gained at 3-years post-randomisation.

For peer review only - http://bmjopen.bmj.com/site/about/guidelines.xhtml

Both deterministic and stochastic sensitivity analyses will be used to address any uncertainty in our estimation of costs, effects and cost-effectiveness. Any uncertainty in the results, based on the stochastic sensitivity analyses, will be illustrated using cost-effectiveness planes and cost-effectiveness acceptability curves. Costs and effects will be discounted at UK recommended rates[14] as they are being estimated beyond a 1-year time horizon.

Radiotherapy Quality Assurance (RT QA): The UK Radiotherapy Trials QA (RTTQA) Group will be responsible for implementing and coordinating the RT QA programme through a named contact for the trial. The programme includes pre-trial and on-trial activities and is outlined below:

Pre-trial QA - Prior to site activation:

• Facility questionnaire – Details of treatment technique, immobilisation, verification and dosimetry will be recorded.

• Dummy Run - All centres must submit a test participant case to the RTTQA group, for review, demonstrating planned radiotherapy treatment to the breast/chest wall, axilla and supraclavicular fossa. The outlining, planning and treatment of this participant case should reflect how the centre intends to plan and treat all participants recruited to POSNOC.

On-trial QA – Ongoing QA during trial recruitment:

• Retrospective individual case reviews – All centres must export radiotherapy plan data to the RTTQA Group for their first ten participants. Data will include participant history, CT data, structure set, plan and dose files. The retrospective review process should include at least three plans with radiotherapy to the nodal region.

• Ongoing data collection - Radiotherapy data related to the RT QA programme will be collected and stored by the RTTQA Group for all participants, regardless of study allocation. A random sample of plans will be reviewed from each site during the trial recruitment period.

The sites will be required to adhere to the POSNOC radiotherapy planning and delivery guidelines when treating participants in the study. The RTTQA Group will promote and monitor compliance to the guidelines for the duration of trial recruitment by systematically reviewing a sample of radiotherapy plans submitted by centres. Radiotherapy guideline deviations, non-compliance or areas of concern will be recorded and if required, discussed with the TMG and individual centres.

Patient and public involvement: As part of the development of the project and protocol, women from the Derby Breast Cancer Support Group, Bosom Buddies (Guildford Breast Cancer Support Group) and Independent Cancer Patients' Voice (ICPV) have commented at all stages on the design and acceptability of the study. The Breast Cancer Care and Lymphoedema Support Network have had the opportunity to contribute to this protocol.

Independent Cancer Patients' Voice is an independent patient advocate group (http://independentcancerpatientsvoice.org.uk/). Two members of ICPV will provide a patient perspective throughout the duration of the study.

For peer review only - http://bmjopen.bmj.com/site/about/guidelines.xhtml

Ethics and dissemination

The study has been approved by the National Research Ethics Service (NRES) Committee East Midlands – Nottingham 2 (REC reference: 13/EM/0459).

The dissemination of the study results will be via a study report and research papers for publication in peer reviewed journals, and presentation at relevant conferences. Reporting will be in compliance with CONSORT recommendations. Publication of the results will be based on outcomes at 5-years following the last recruited participant.

A summary of the results will be made available to participants through a newsletter (unless they state they do not wish to receive this), and will also be publicised through Independent Cancer Patients' Voice, Cancer Research UK, Breast Cancer Now and Lymphoedema Support Network.

Trial status: Recruiting. On 09 June 2021, 1866 patients have been included.

Data sharing plan: Requests for data collected for POSNOC will be considered by the TMG and NCTU Data Sharing review panel.

Additional Documentation: The current full trial protocol (v 6.0 26 March 2020), details of previous protocol amendments, copies of current PIL and informed consent forms, oversight committee charters and a full list of participating sites can be found at www.posnoc.co.uk.

Author contributions

Conceptualization and writing original protocol: AG conceived the study and prepared the first draft of the original protocol.

Funding acquisition (UK and ANZ): AG, BM, LF, LD, MWR, DD, RC, AF, RGN, VJ, DW, MC, DW, VK, IE, PF

Trial design and methodology: All authors contributed to study design and methodology.

Statistics: AF, RGN, AM, WT

Review and editing: All authors read, critically revised and approved the manuscript before submission.

Radiotherapy QA: DD, DM, ZN, PS, AC, EM

Trial management and co-ordination: All authors are current or past TMG members and contributed to trial management and co-ordination.

For peer review only

For peer review only - http://bmjopen.bmj.com/site/about/guidelines.xhtml

Footnotes

Trial Management Group members: Amit Goyal (Chief Investigator and Chair), Shabina Sadiq, Mickey Lewis, Rachel Haines, Alan Montgomery, Wei Tan, G Bruce Mann, Kathryn Monson, Valerie Jenkins, Patricia Fairbrother, David Dodwell, Tara Homer, Heath Badger, Zohal Nabi, Romaana Mir, Elizabeth Miles, Luke Vale, Malcolm W Reed, Lesley Fallowfield, Robert Coleman, Vaughan Keeley, Ian Ellis, Daniel Davis, Teresa Grieve (past members: Lelia Duley, Diane Whitham, Margaret Childs, Apostolos Fakis, Robert G Newcombe, David Whynes, Eleanor Mitchell, Rebecca Haydock, Clare Brittain, Clare Upton, Sarah Craig)

Nottingham Clinical Trials Unit staff: Alan Montgomery, Rachel Haines, Shabina Sadiq, Wei Tan, Mickey Lewis, Aisha Shafayat, Charlotte Gidman, Mara Ozolins, Lelia Duley, Diane Whitham, Margaret Childs, Eleanor Mitchell, Rebecca Haydock, Clare Brittain, Clare Upton, Sarah Craig

Patient Reported Outcomes staff (Sussex Health Outcomes Research & Education in Cancer (SHORE-C)): Kathryn Monson, Valerie Jenkins, Lesley Fallowfield

Health Economics staff (Newcastle University): Tara Homer, Luke Vale (past staff: Laura Ternent)

Breast Cancer Trials Australia and New Zealand staff: Heath Badger, Bruce Mann, Nicole Francis, Flonda Probert, Lisa Paksec, Rose Lucas (past staff: Annette Dempsey, Rochelle Thornton)

Radiotherapy Quality Assurance team: Daniel Megias, Zohal Nabi, Preetinder Singh, Andrei Caraman, Elizabeth Miles, Romaana Mir, Roeum Butt, Hannah Price

Independent Trial Steering Committee members: Alastair Thompson (Chair), Alistair Ring, Nisha Sharma, Peter Barrett Lee, Julie Wolfarth, Ian White, Ramsey Cutress (past members:

Independent Data Monitoring Committee members: Judith Bliss (chair), Daniel Rea, Peter Dubsky

Sponsor: The trial is sponsored by the University Hospitals of Derby and Burton NHS Foundation Trust (uhdb.sponsor@nhs.net). Sponsor staff - Daniel Davis, Joanne Thornhill, Anne Shaw, Amy Farmer, Teresa Grieve

Acknowledgments: We thank the patients who are participating in this trial, and staff at the participating centres and the independent data monitoring committee and trial steering committee for overseeing the trial.

Funding: This project is funded by the National Institute for Health Research Health Technology Assessment Programme (project number 12/35/17). Australian and New Zealand participation is funded by National Health and Medical Research Council (NHMRC) (project grant 1083172).

Disclaimer: The views expressed are those of the authors and not necessarily those of the Health Technology Assessment Programme, NIHR, NHS or the Department of Health and Social Care.

For peer review only - http://bmjopen.bmj.com/site/about/guidelines.xhtml

Competing interests: LV was a member of the NIHR Health Technology Assessment Programme, Clinical Trials and Evaluation Panel from 2014-2018.

Reference List

1. Buchholz, T.A., et al., *Impact of systemic treatment on local control for patients with lymph node-negative breast cancer treated with breast-conservation therapy.* J.Clin.Oncol., 2001. **19**(8): p. 2240-2246.
2. Mansel, R.E., et al., *Results of a national training programme in sentinel lymph node biopsy for breast cancer.* Br J Surg, 2013. **100**(5): p. 654-61.
3. Khonji, N.I., et al., *Incidence of nodes in completion mastectomy specimens following breast conservation and axillary clearance.* Br.J.Surg., 2002. **89**(10): p. 1294-1295.
4. Giuliano, A.E., et al., *Locoregional recurrence after sentinel lymph node dissection with or without axillary dissection in patients with sentinel lymph node metastases: the American College of Surgeons Oncology Group Z0011 randomized trial.* Ann Surg, 2010. **252**(3): p. 426-32; discussion 432-3.
5. Azghadi, S., M. Daly, and J. Mayadev, *Practice Patterns of Radiation Field Design for Sentinel Lymph Node-Positive Early-Stage Breast Cancer.* Clin.Breast Cancer, 2016. **16**(5): p. 410-417.
6. Donker, M., et al., *Radiotherapy or surgery of the axilla after a positive sentinel node in breast cancer (EORTC 10981-22023 AMAROS): a randomised, multicentre, open-label, phase 3 non-inferiority trial.* Lancet Oncol, 2014. **15**(12): p. 1303-1310.
7. Armer, J.M., et al., *Predicting breast cancer-related lymphedema using self-reported symptoms.* Nurs.Res., 2003. **52**(6): p. 370-379.
8. Kennedy, C.A., et al., *Measurement properties of the QuickDASH (Disabilities of the Arm, Shoulder and Hand) outcome measure and cross-cultural adaptations of the QuickDASH: a systematic review.* Qual.Life Res., 2013.
9. Coster, S., K. Poole, and L.J. Fallowfield, *The validation of a quality of life scale to assess the impact of arm morbidity in breast cancer patients post-operatively.* Breast Cancer Res.Treat., 2001. **68**(3): p. 273-282.
10. Spielberger, C., *The Manual for the State-Trait Inventory.* Palo Alto, CA: Consulting Psychologists Press Inc., 1983.
11. Sanghani, M., E.M. Balk, and B. Cady, *Impact of axillary lymph node dissection on breast cancer outcome in clinically node negative patients: a systematic review and meta-analysis.* Cancer, 2009. **115**(8): p. 1613-20.
12. Miettinen, O. and M. Nurminen, *Comparative analysis of two rates.* Stat Med, 1985. **4**(2): p. 213-26.
13. Matthews, J.N., et al., *Analysis of serial measurements in medical research.* BMJ, 1990. **300**(6719): p. 230-235.
14. *National Institute for Health and Care Excellence (NICE): Guide to the methods of technology appraisal 2013.* https://www.nice.org.uk/article/pmg9, 2013.

For peer review only - http://bmjopen.bmj.com/site/about/guidelines.xhtml

Figure 1. Trial flow chart

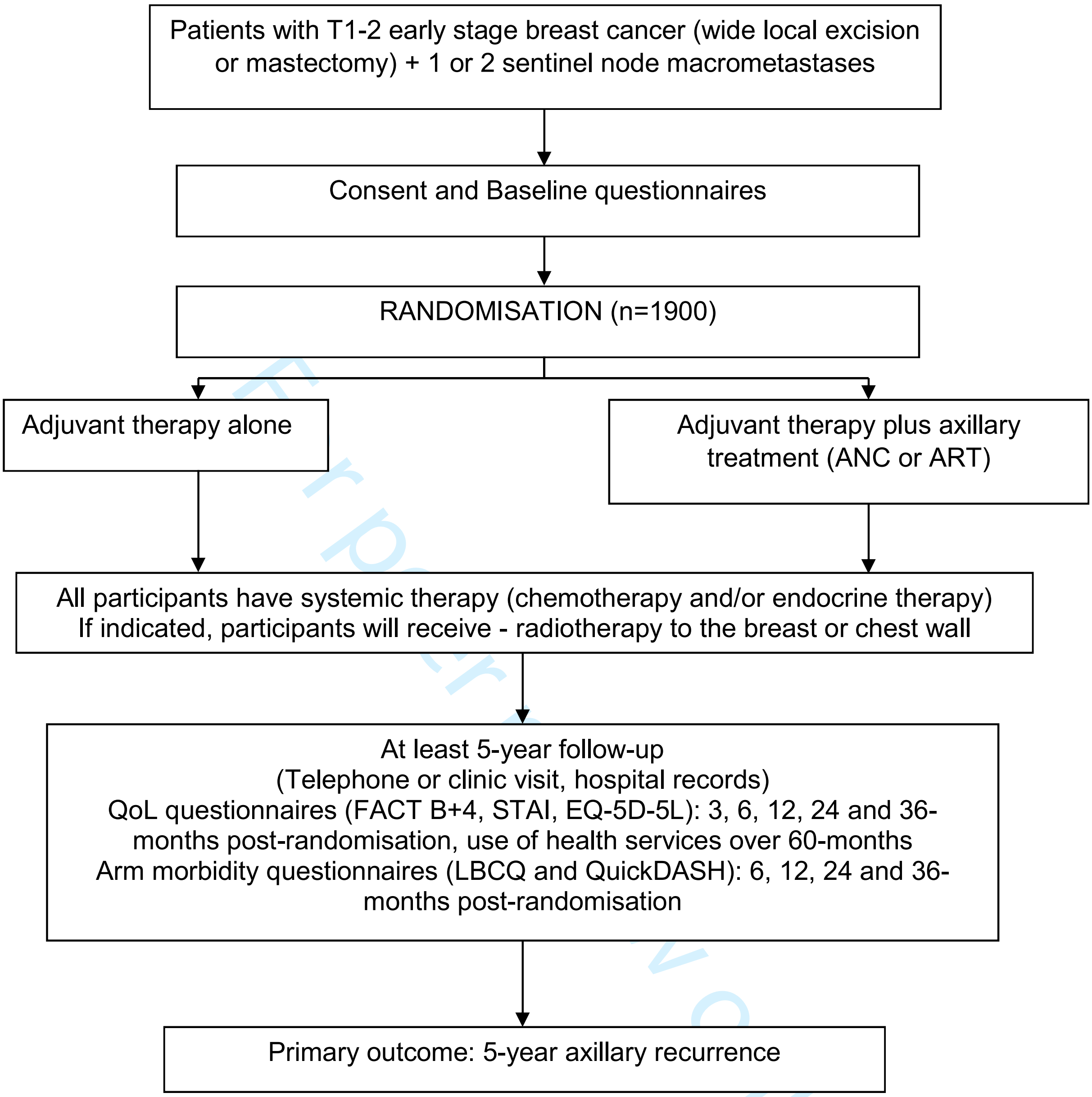

ANC, axillary node clearance; ART axillary radiotherapy; FACT B+4, Functional Assessment of Cancer Therapy – Breast +4; STAI, State-Trait Anxiety Inventory; LBCQ, Lymphoedema and Breast Cancer Questionnaire; QuickDASH, Disabilities of the Arm, Shoulder and Hand questionnaire.

For peer review only - http://bmjopen.bmj.com/site/about/guidelines.xhtml

SPIRIT

STANDARD PROTOCOL ITEMS: RECOMMENDATIONS FOR INTERVENTIONAL TRIALS

SPIRIT 2013 Checklist: Recommended items to address in a clinical trial protocol and related documents*

Section/item	Item No	Description	Addressed on page number
**Administrative information**			
Title	1	Descriptive title identifying the study design, population, interventions, and, if applicable, trial acronym	1
Trial registration	2a	Trial identifier and registry name. If not yet registered, name of intended registry	2
	2b	All items from the World Health Organization Trial Registration Data Set	throughout
Protocol version	3	Date and version identifier	13
Funding	4	Sources and types of financial, material, and other support	14
Roles and responsibilities	5a	Names, affiliations, and roles of protocol contributors	1, 13-14
	5b	Name and contact information for the trial sponsor	14
	5c	Role of study sponsor and funders, if any, in study design; collection, management, analysis, and interpretation of data; writing of the report; and the decision to submit the report for publication, including whether they will have ultimate authority over any of these activities	14
	5d	Composition, roles, and responsibilities of the coordinating centre, steering committee, endpoint adjudication committee, data management team, and other individuals or groups overseeing the trial, if applicable (see Item 21a for data monitoring committee)	13-14

For peer review only

Introduction

Background and rationale	6a	Description of research question and justification for undertaking the trial, including summary of relevant studies (published and unpublished) examining benefits and harms for each intervention	4
	6b	Explanation for choice of comparators	4
Objectives	7	Specific objectives or hypotheses	5
Trial design	8	Description of trial design including type of trial (eg, parallel group, crossover, factorial, single group), allocation ratio, and framework (eg, superiority, equivalence, noninferiority, exploratory)	5

Methods: Participants, interventions, and outcomes

Study setting	9	Description of study settings (eg, community clinic, academic hospital) and list of countries where data will be collected. Reference to where list of study sites can be obtained	5
Eligibility criteria	10	Inclusion and exclusion criteria for participants. If applicable, eligibility criteria for study centres and individuals who will perform the interventions (eg, surgeons, psychotherapists)	5-6
Interventions	11a	Interventions for each group with sufficient detail to allow replication, including how and when they will be administered	6
	11b	Criteria for discontinuing or modifying allocated interventions for a given trial participant (eg, drug dose change in response to harms, participant request, or improving/worsening disease)	n/a
	11c	Strategies to improve adherence to intervention protocols, and any procedures for monitoring adherence (eg, drug tablet return, laboratory tests)	n/a
	11d	Relevant concomitant care and interventions that are permitted or prohibited during the trial	6
Outcomes	12	Primary, secondary, and other outcomes, including the specific measurement variable (eg, systolic blood pressure), analysis metric (eg, change from baseline, final value, time to event), method of aggregation (eg, median, proportion), and time point for each outcome. Explanation of the clinical relevance of chosen efficacy and harm outcomes is strongly recommended	6-7
Participant timeline	13	Time schedule of enrolment, interventions (including any run-ins and washouts), assessments, and visits for participants. A schematic diagram is highly recommended (see Figure)	Figure 1, Table 1

For peer review only - http://bmjopen.bmj.com/site/about/guidelines.xhtml

Sample size	14	Estimated number of participants needed to achieve study objectives and how it was determined, including clinical and statistical assumptions supporting any sample size calculations	10
Recruitment	15	Strategies for achieving adequate participant enrolment to reach target sample size	9
**Methods: Assignment of interventions (for controlled trials)**			
Allocation:			
Sequence generation	16a	Method of generating the allocation sequence (eg, computer-generated random numbers), and list of any factors for stratification. To reduce predictability of a random sequence, details of any planned restriction (eg, blocking) should be provided in a separate document that is unavailable to those who enrol participants or assign interventions	5
Allocation concealment mechanism	16b	Mechanism of implementing the allocation sequence (eg, central telephone; sequentially numbered, opaque, sealed envelopes), describing any steps to conceal the sequence until interventions are assigned	5
Implementation	16c	Who will generate the allocation sequence, who will enrol participants, and who will assign participants to interventions	9
Blinding (masking)	17a	Who will be blinded after assignment to interventions (eg, trial participants, care providers, outcome assessors, data analysts), and how	n/a
	17b	If blinded, circumstances under which unblinding is permissible, and procedure for revealing a participant's allocated intervention during the trial	n/a
**Methods: Data collection, management, and analysis**			
Data collection methods	18a	Plans for assessment and collection of outcome, baseline, and other trial data, including any related processes to promote data quality (eg, duplicate measurements, training of assessors) and a description of study instruments (eg, questionnaires, laboratory tests) along with their reliability and validity, if known. Reference to where data collection forms can be found, if not in the protocol	9-10
	18b	Plans to promote participant retention and complete follow-up, including list of any outcome data to be collected for participants who discontinue or deviate from intervention protocols	9-10

Data management	19	Plans for data entry, coding, security, and storage, including any related processes to promote data quality (eg, double data entry; range checks for data values). Reference to where details of data management procedures can be found, if not in the protocol	9-10
Statistical methods	20a	Statistical methods for analysing primary and secondary outcomes. Reference to where other details of the statistical analysis plan can be found, if not in the protocol	10-12
	20b	Methods for any additional analyses (eg, subgroup and adjusted analyses)	10-12
	20c	Definition of analysis population relating to protocol non-adherence (eg, as randomised analysis), and any statistical methods to handle missing data (eg, multiple imputation)	10-12
**Methods: Monitoring**			
Data monitoring	21a	Composition of data monitoring committee (DMC); summary of its role and reporting structure; statement of whether it is independent from the sponsor and competing interests; and reference to where further details about its charter can be found, if not in the protocol. Alternatively, an explanation of why a DMC is not needed	14
	21b	Description of any interim analyses and stopping guidelines, including who will have access to these interim results and make the final decision to terminate the trial	n/a
Harms	22	Plans for collecting, assessing, reporting, and managing solicited and spontaneously reported adverse events and other unintended effects of trial interventions or trial conduct	9
Auditing	23	Frequency and procedures for auditing trial conduct, if any, and whether the process will be independent from investigators and the sponsor	9
**Ethics and dissemination**			
Research ethics approval	24	Plans for seeking research ethics committee/institutional review board (REC/IRB) approval	13
Protocol amendments	25	Plans for communicating important protocol modifications (eg, changes to eligibility criteria, outcomes, analyses) to relevant parties (eg, investigators, REC/IRBs, trial participants, trial registries, journals, regulators)	13

Consent or assent	26a	Who will obtain informed consent or assent from potential trial participants or authorised surrogates, and how (see Item 32)	5
	26b	Additional consent provisions for collection and use of participant data and biological specimens in ancillary studies, if applicable	n/a
Confidentiality	27	How personal information about potential and enrolled participants will be collected, shared, and maintained in order to protect confidentiality before, during, and after the trial	10
Declaration of interests	28	Financial and other competing interests for principal investigators for the overall trial and each study site	14
Access to data	29	Statement of who will have access to the final trial dataset, and disclosure of contractual agreements that limit such access for investigators	13
Ancillary and post-trial care	30	Provisions, if any, for ancillary and post-trial care, and for compensation to those who suffer harm from trial participation	13
Dissemination policy	31a	Plans for investigators and sponsor to communicate trial results to participants, healthcare professionals, the public, and other relevant groups (eg, via publication, reporting in results databases, or other data sharing arrangements), including any publication restrictions	13
	31b	Authorship eligibility guidelines and any intended use of professional writers	13
	31c	Plans, if any, for granting public access to the full protocol, participant-level dataset, and statistical code	13
**Appendices**			
Informed consent materials	32	Model consent form and other related documentation given to participants and authorised surrogates	13
Biological specimens	33	Plans for collection, laboratory evaluation, and storage of biological specimens for genetic or molecular analysis in the current trial and for future use in ancillary studies, if applicable	n/a

*It is strongly recommended that this checklist be read in conjunction with the SPIRIT 2013 Explanation & Elaboration for important clarification on the items. Amendments to the protocol should be tracked and dated. The SPIRIT checklist is copyrighted by the SPIRIT Group under the Creative Commons "Attribution-NonCommercial-NoDerivs 3.0 Unported" license.

For peer review only - http://bmjopen.bmj.com/site/about/guidelines.xhtml

BMJ Open

Protocol: POSNOC - Positive Sentinel Node: adjuvant therapy alone versus adjuvant therapy plus Clearance or axillary radiotherapy. A randomised controlled trial of axillary treatment in women with early stage breast cancer who have metastases in one or two sentinel nodes

Journal:	*BMJ Open*
Manuscript ID	bmjopen-2021-054365.R2
Article Type:	Protocol
Date Submitted by the Author:	28-Oct-2021
Complete List of Authors:	Goyal, Amit; Royal Derby Hospital,<br>Mann, G. Bruce; The University of Melbourne<br>Fallowfield, Lesley; University of Sussex, SHORE-C, Brighton & Sussex Medical School<br>Duley, Lelia; Nottingham Clinical Trials Unit, University of Nottingham<br>Reed, Malcolm; Brighton and Sussex Medical School,<br>Dodwell, David; Oxford University, Nuffield Department of Population Health<br>Coleman, Robert E.; Weston Park Hospital<br>Fakis, Apostolos; Royal Derby Hospital, Research and Development<br>Newcombe, Robert; Cardiff University, Department of Primary Care and Public Health<br>Jenkins, Valerie; University of Sussex,<br>Whitham, Diane; The University of Nottingham, Clinical Trials Unit<br>Childs, Margo; University of Nottingham,<br>Whynes, David; University of Nottingham<br>Keeley, Vaughn; University of Nottingham,<br>Ellis, Ian ; University of Nottingham, Division of Oncology, School of Medicine<br>Fairbrother, Patricia; Independent Cancer Patients' Voice,<br>Sadiq, Shabina; Nottingham Clinical Trials Unit, University of Nottingham<br>Monson, Kathryn; Brighton & Sussex Medical School, Sussex Health Outcomes, Research & Education in Cancer (SHORE-C)<br>Montgomery, Alan; Nottingham Clinical Trials Unit, University of Nottingham<br>Tan, Wei; University of Nottingham School of Medecine, Nottingham Clinical Trials Unit<br>Vale, Luke; Newcastle University, Health Economics Group, Population Health Sciences Institute<br>Homer, Tara; Newcastle University, Health Economics Group, Population Health Sciences Institute<br>Badger, Heath; Breast Cancer Trials<br>Haines, Rachel; University of Nottingham School of Medecine, Nottingham Clinical Trials Unit<br>Lewis, Mickey; Nottingham Clinical Trials Unit, University of Nottingham<br>Megias, Daniel; National Radiotherapy Trials Quality Assurance Group, Mount Vernon Cancer Centre

	Nabi, Zohal; National Radiotherapy Trials Quality Assurance Group, Mount Vernon Cancer Centre<br>Singh, Preetinder; National Radiotherapy Trials Quality Assurance Group, Mount Vernon Cancer Centre<br>Caraman, Andrei; National Radiotherapy Trials Quality Assurance Group, Mount Vernon Cancer Centre<br>Miles, Elizabeth; National Radiotherapy Trials Quality Assurance Group, Mount Vernon Hospital

**Primary Subject Heading**:	Surgery
Secondary Subject Heading:	Surgery
Keywords:	Breast surgery < SURGERY, Breast tumours < ONCOLOGY, Adult radiotherapy < RADIOTHERAPY

SCHOLARONE™
Manuscripts

I, the Submitting Author has the right to grant and does grant on behalf of all authors of the Work (as defined in the below author licence), an exclusive licence and/or a non-exclusive licence for contributions from authors who are: i) UK Crown employees; ii) where BMJ has agreed a CC-BY licence shall apply, and/or iii) in accordance with the terms applicable for US Federal Government officers or employees acting as part of their official duties; on a worldwide, perpetual, irrevocable, royalty-free basis to BMJ Publishing Group Ltd ("BMJ") its licensees and where the relevant Journal is co-owned by BMJ to the co-owners of the Journal, to publish the Work in this journal and any other BMJ products and to exploit all rights, as set out in our licence.

The Submitting Author accepts and understands that any supply made under these terms is made by BMJ to the Submitting Author unless you are acting as an employee on behalf of your employer or a postgraduate student of an affiliated institution which is paying any applicable article publishing charge ("APC") for Open Access articles. Where the Submitting Author wishes to make the Work available on an Open Access basis (and intends to pay the relevant APC), the terms of reuse of such Open Access shall be governed by a Creative Commons licence – details of these licences and which Creative Commons licence will apply to this Work are set out in our licence referred to above.

Other than as permitted in any relevant BMJ Author's Self Archiving Policies, I confirm this Work has not been accepted for publication elsewhere, is not being considered for publication elsewhere and does not duplicate material already published. I confirm all authors consent to publication of this Work and authorise the granting of this licence.

For peer review only

POSNOC protocol BMJ open v 2.0

Title: Protocol: POSNOC - POsitive Sentinel NOde: adjuvant therapy alone versus adjuvant therapy plus Clearance or axillary radiotherapy. A randomised controlled trial of axillary treatment in women with early stage breast cancer who have metastases in one or two sentinel nodes

Authors: Amit Goyal[1], G Bruce Mann[2], Lesley Fallowfield[3], Lelia Duley[4], Malcolm W Reed[5], David Dodwell[6], Robert Coleman[7], Apostolos Fakis[1], Robert G Newcombe[8], Valerie Jenkins[3], Diane Whitham[4], Margo Childs[4], David Whynes[4], Vaughan Keeley[1], Ian Ellis[9], Patricia Fairbrother[10], Shabina Sadiq[4], Kathryn Monson[3], Alan A Montgomery[4], Wei Tan[4], Luke Vale[11], Tara Homer[11], Heath Badger[12], Rachel Haines[4], Mickey Lewis[4], Daniel Megias[13], Zohal Nabi[13], Preetinder Singh[13], Andrei Caraman[13], Elizabeth Miles[13] on behalf of the POSNOC Trial Management Group

Institutions: [1]University Hospitals of Derby and Burton NHS Foundation Trust, Derby, UK; [2]Victorian Comprehensive Cancer Centre, Melbourne, Australia; [3]SHORE-C, University of Sussex, Brighton, UK; [4]Nottingham Clinical Trials Unit, University of Nottingham, Nottingham, UK; [5]Brighton and Sussex Medical School, University of Sussex, Brighton, UK; [6]Nuffield Department of Population Health, University of Oxford, Oxford, UK; [7]Weston Park Hospital, Sheffield, UK; [8]Cardiff University, Cardiff, UK; [9]University of Nottingham, Nottingham, UK; [10]Independent Cancer Patients' Voice, London, UK; [11]Newcastle University, Newcastle, UK; [12]Breast Cancer Trials, Newcastle, Australia; [13]National Radiotherapy Trials QA (RTTQA) Group, Mount Vernon Cancer Centre, Northwood, UK

Correspondence: Amit Goyal (Chief Investigator), University Hospitals of Derby and Burton NHS Foundation Trust, Derby, UK. Email: amit.goyal@nhs.net

Tel: +44(0)1332 785538, Twitter handle: @_amit_goyal

Word count: 4000

Figures: 1

Tables: 1

For peer review only

Abstract

Introduction

ACOSOG-Z0011(Z11) trial showed that axillary node clearance(ANC) may be omitted in women with ≤2 positive nodes undergoing breast conserving surgery (BCS) and whole breast radiotherapy (RT). A confirmatory study is needed to clarify the role of axillary treatment in women with ≤2 macrometastases undergoing BCS and groups that were not included in Z11 e.g. mastectomy and those with microscopic extranodal invasion.

The primary objective of POSNOC is to evaluate whether for women with breast cancer and 1 or 2 macrometastases, adjuvant therapy alone is non-inferior to adjuvant therapy plus axillary treatment, in terms of 5-year axillary recurrence.

Methods and analysis

POSNOC is a pragmatic, multicentre, non-inferiority, international trial with participants randomised in a 1:1 ratio. Women are eligible if they have T1/T2, unifocal or multifocal invasive breast cancer, and 1 or 2 macrometastases at sentinel node biopsy, with or without extranodal extension. In the intervention group women receive adjuvant therapy alone, in the standard care group they receive ANC or axillary RT. In both groups women receive adjuvant therapy, according to local guidelines. This includes systemic therapy and, if indicated, RT to breast or chest wall. The UK Radiotherapy Trials Quality Assurance group manages the in-built Radiotherapy Quality Assurance programme. Primary endpoint is 5-year axillary recurrence. Secondary outcomes are arm morbidity assessed by LBCQ and QuickDASH questionnaires; Quality of Life and anxiety as assessed with FACT B+4 and STAI questionnaires respectively; other oncological outcomes; economic evaluation using EQ-5D-5L. Target sample size is 1900. Primary analysis is per protocol. Recruitment started on 1 August 2014 and as of 09 June 2021, 1866 participants have been randomised.

Ethics and dissemination: Protocol was approved by the National Research Ethics Service Committee East Midlands – Nottingham 2 (REC reference: 13/EM/0459). Results will be submitted for publication in peer-reviewed journals.

Trial registration number: ISRCTN54765244; NCT02401685

Strengths and limitations of this study

- POSNOC includes women undergoing mastectomy and those with extranodal invasion
- Only women with 1 or 2 macrometastases are eligible for randomisation

For peer review only - http://bmjopen.bmj.com/site/about/guidelines.xhtml

- POSNOC has an in-built radiotherapy quality assurance programme co-ordinated by the UK Radiotherapy Trials QA (RTTQA) Group
- POSNOC is an international trial - UK, Australia and New Zealand

Key words: POSNOC, sentinel node biopsy, breast cancer, axillary node clearance, axillary radiotherapy

For peer review only

For peer review only - http://bmjopen.bmj.com/site/about/guidelines.xhtml

Introduction

Currently, women with early breast cancer and 1 or 2 macrometastases in sentinel lymph nodes (SNs) generally undergo some form of axillary treatment, either surgical axillary node clearance (ANC) or radiotherapy to the axilla (ART). The use of axillary treatment is based on the assumption that it reduces the risk of axillary recurrence and might improve survival. However, axillary treatment damages lymphatic drainage from the arm and women can subsequently develop lymphoedema, restricted shoulder movement, pain, numbness, and other sensory problems. These adverse effects interfere with daily activities, are distressing, impair quality of life (QoL) and are costly to the NHS in terms of rehabilitative treatments, as they are often permanent and symptom relief is difficult.

Axillary treatment may no longer be necessary for this group of women, as they usually have a low axillary tumour burden and current systemic therapy is effective at preventing breast and axillary recurrence[1]. Also, sentinel node biopsy has already removed the lymph nodes most likely to have metastasis[2]. Moreover, when adjuvant therapy includes radiotherapy to the breast or chest wall, the lower axilla may be treated as it is often included in the tangential irradiation field, and some lower level axillary nodes may be removed at mastectomy[3].

This hypothesis is supported by the Z11 trial[4] that showed that ANC may be omitted in women with ≤2 positive nodes undergoing breast conserving surgery (BCS) and receiving whole breast RT. Axillary recurrence was low, and there were no clear differences between the two groups (ANC 0.5% vs. no ANC 0.9%) at 6.3 years. Z11 has not had the desired impact on change of clinical practice because of several limitations. The study did not meet its recruitment goal, approximately 40% of participants had micrometastases, many were lost to follow-up (19.4%), and there was a lack of radiation therapy quality assurance. Most US radiation oncologists continue to treat the undissected axilla in women with micro or macro metastases with ART[5]. Therefore, it appears that clinicians in the US are interpreting Z11 in line with AMAROS study which showed that ART is a less morbid alterative to axillary lymph node dissection (ALND) in women with low volume nodal disease[6] rather than omitting axillary treatment. Furthermore, the results are not applicable to subgroups that were not included such as those with mastectomy and microscopic extranodal invasion.

For peer review only - http://bmjopen.bmj.com/site/about/guidelines.xhtml

Objective

The primary objective of the POSNOC trial is to evaluate whether, for women with early breast cancer and one or two nodes with macrometastases, adjuvant therapy alone is non-inferior to adjuvant therapy plus axillary treatment (ANC or ART), in terms of 5-year axillary recurrence.

Methods and analysis

Design: POSNOC is a pragmatic randomised, multicentre, non-inferiority, international (UK, Australia, New Zealand) trial. Target sample size is 1900 participants, with 5-year follow-up. Flow of participants through the trial is summarised in Figure 1.

Randomisation: Participants are allocated via a secure internet-based system, using minimisation that is weighted toward minimising imbalance between trial arms with probability 0.8. Allocation is stratified by recruiting site and minimised by the following variables: age (<50, ≥50 years); type of breast surgery (breast-conserving surgery (BCS), mastectomy); estrogen receptor status (ER) (positive, negative); number of positive nodes (1, 2); intra-operative sentinel assessment using OSNA (yes, no).

Target population: Women with unifocal or multifocal invasive breast cancer with the largest lesion ≤5cm, who have 1 or 2 sentinel nodes with macrometastases (>2mm), with or without extranodal invasion.

Women are usually screened for eligibility after their initial breast surgery and sentinel node biopsy. In hospitals where sentinel node histology is assessed intra-operatively, women are asked to give their consent pre-operatively and then randomised intra-operatively if their sentinel nodes are found to be positive.

Inclusion Criteria: Women are eligible for inclusion if ALL of the following criteria apply:

- 18 years or older
- Unifocal or multi-focal invasive tumour ≤5 cm in its largest dimension, measured pathologically or largest invasive tumour diameter on radiology for women who are randomised intra-operatively or who undergo sentinel node biopsy before neoadjuvant therapy (tumour size is based only on the single largest tumour)
- At sentinel node biopsy have 1 or 2 nodes with macrometastases (tumour deposit >2.0mm in largest dimension or defined as macrometastases on molecular assay)
- Fit for axillary treatment and adjuvant therapy
- Have given informed consent

Exclusion Criteria: Women are excluded if they have any of the following:

For peer review only - http://bmjopen.bmj.com/site/about/guidelines.xhtml

- Bilateral invasive breast cancer
- more than 2 nodes with macrometastases
- neoadjuvant therapy for breast cancer except
  - o if sentinel node biopsy performed prior to neoadjuvant therapy in women with early breast cancer
  - o short duration of neoadjuvant endocrine therapy (up to 3-months)
- previous axillary surgery on the same body side as the scheduled sentinel node biopsy
- not planned to receive adjuvant systemic therapy
- previous cancer less than 5-years previously or concomitant malignancy except
  - o basal or squamous cell carcinoma of the skin
  - o in situ carcinoma of the cervix
  - o in situ melanoma
  - o contra- or ipsilateral in situ breast cancer

Trial treatment arms: All participants receive adjuvant therapy according to local guidelines. This includes chemotherapy and/or endocrine therapy for all women, and radiotherapy to breast or chest wall if indicated. Human epidermal growth factor receptor 2 (HER2) targeted treatment is administered when indicated.

Trial participants are allocated to one of the following two groups:

(i) Adjuvant therapy alone (intervention group)

See above for adjuvant therapy. Axillary and supraclavicular fossa radiotherapy is not allowed.

(ii) Adjuvant therapy plus axillary treatment (standard care group)

See above for adjuvant therapy. Axillary treatment can be axillary node clearance or axillary radiotherapy as per local guidelines.

Outcomes

Primary Outcome: The primary outcome is 5-year axillary recurrence defined as pathologically (cytology or biopsy) and/or radiologically confirmed recurrence in lymph nodes draining the primary tumour site.

Secondary Outcomes (Table 1)**:**

- Arm morbidity assessed by a) Lymphoedema and Breast Cancer Questionnaire (LBCQ)[7] - the definition of lymphoedema is 'yes' to the two questions -'heaviness during the past year' and 'swelling now'; b) QuickDASH questionnaire[8] - Shoulder, arm and hand disability is defined as a change from baseline in the QuickDASH score of at least 14 points.

For peer review only - http://bmjopen.bmj.com/site/about/guidelines.xhtml

- QoL assessed by Functional Assessment of Cancer Therapy-Breast+4 (FACT B+4) questionnaire[9]. The total FACT B+4 score reflects global QoL and comprises physical (7 items), functional (7 items), social (7 items) and emotional well-being (6 items) plus the breast cancer concerns and arm morbidity scale (14 items) (total of 41 items; total score ranges from 0-164). The 5 item arm morbidity subscale score comprises the sum of the scores from items B3, B10, B11, B12, B13 (range 0-20) and will be analysed separately. Analyses will include the proportion of participants in the two allocated treatment groups reporting 'somewhat', 'quite a bit' and 'very much' for each of the 5 items.
- Anxiety assessed by using the Spielberger State/Trait Anxiety Inventory(STAI)[10]. The STAI consists of 2 questionnaires with 20 items. It assesses anxiety proneness (Trait) and the current state of anxiety or anxiety change (State). Each item is rated on a four point Likert scale. High STAI scores signify greater anxiety. The Trait anxiety is measured only once and the State at each time point.
- Economic outcomes will include costs to the NHS, quality-adjusted life years (QALYs) based on responses to the EQ-5D-5L, and the primary outcome, axillary recurrence. For more details, see Economic Analysis section below.
- Local (breast or chest wall) recurrence; defined as pathologically (cytology or biopsy) and/or radiologically confirmed recurrence after mastectomy in the skin or soft tissue of the chest wall within the anatomical area bounded by the mid-sternal line, the clavicle, the posterior axillary line and the costal margin or any type of breast carcinoma in the breast after conservation therapy.
- Regional (nodal) recurrence; defined as pathologically (cytology or biopsy) and/or radiologically confirmed recurrent tumour in the lymph nodes in the ipsilateral axilla, infraclavicular, supraclavicular fossa, interpectoral area or ipsilateral internal mammary chain.
- Distant metastasis; defined as confirmed metastasis (positive pathology and/or definitive evidence on imaging) in all other sites of recurrence and may include those classified as: soft-tissue category, visceral category, central nervous system and skeletal spread.
- Time to axillary recurrence is the time between the date of randomisation and the date of axillary recurrence, measured in days.

For peer review only

For peer review only - http://bmjopen.bmj.com/site/about/guidelines.xhtml

- Axillary recurrence free survival is the time between the date of randomisation and date of confirmed axillary recurrence or date of death, whichever comes first, measured in days.
- Disease free survival; defined as the time between the date of randomisation and the date of disease progression (i.e. local or regional recurrence or distant metastasis) or death, whichever comes first, measured in days.
- Overall survival is the time between the date of randomisation and the date of death from any cause.
- Contralateral breast cancer is new primary malignancy in the opposite breast unless obviously contiguous with recurrent chest wall disease or proven on cytology/biopsy to be of metastatic origin.
- Non-breast cancer is any new non-breast primary malignancy, except for adequately treated, superficial squamous or basal cell carcinoma of the skin, or carcinoma in situ of the cervix.

Table 1. Secondary outcomes are assessed at the following time points:

Secondary Outcome	Assessment time point (months)						
	3	6	12	24	36	48	60
Arm morbidity		X	X	X	X		
Quality of life	X	X	X	X	X		
Anxiety	X	X	X	X	X		
NHS costs	X	X	X	X	X	X	X
EQ-5D-5L	X	X	X	X	X		
Incremental cost per QALY gained					X		
Incremental cost per reduction in axillary recurrence							X
Local (breast or chest wall) recurrence		X	X	X	X	X	X
Regional (nodal) recurrence		X	X	X	X	X	X
Distant metastasis		X	X	X	X	X	X
Time to axillary recurrence		X	X	X	X	X	X
Axillary recurrence free survival		X	X	X	X	X	X
Disease free survival		X	X	X	X	X	X

For peer review only - http://bmjopen.bmj.com/site/about/guidelines.xhtml

Overall survival		X	X	X	X	X	X
Contralateral breast cancer		X	X	X	X	X	X
Non-breast malignancy		X	X	X	X	X	X

Participant Identification: Potential trial participants are identified at multi-disciplinary meetings. A participant information leaflet (PIL) and video is given to the participant

Based on local hospital practices, there are four participant pathways for recruitment:

(i) After primary breast surgery: Women who have a positive sentinel node are approached with regard to trial participation at the first post-operative clinic visit to offer trial participation.

(ii) Before primary breast surgery: In some hospitals sentinel node assessment is performed during primary breast surgery. In these hospitals, women are approached about the trial before surgery. If they are willing to participate consent is obtained prior to surgery. Randomisation is intra-operative if the SNs are confirmed as positive.

(iii) Post-chemotherapy: Women may be approached about the trial during and after chemotherapy but before starting axillary treatment (ANC or ART).

(iv) SNB prior to neoadjuvant therapy: Where the sentinel node biopsy is performed prior to neoadjuvant therapy patients can be approached during or after neoadjuvant therapy but prior to axillary treatment (ANC or ART).

Study Assessments

- **Baseline:** Baseline data are collected from the participant's medical notes and questionnaires.
- **Follow-up**: Follow-up assessments are at 6, 12, 24, 36, 48 and 60-months post randomisation with an additional postal questionnaire at 3-months.

The 6, 12, 24, 36, 48 and 60 month follow-up is in person or over the telephone, according to local practice. If contact cannot be made with the participant then medical notes can be used.

Data Collection: Trial data are entered at site into electronic case report forms (eCRF). Participants are identified only by their unique participant ID number. Access to the database is restricted and secure.

Data from routine medical notes can be used to collect outcomes for those who have been lost to follow-up or no longer wish to attend research visits.

Monitoring of Quality and Safety: Data handling and trial monitoring procedures are detailed in trial-specific data management and trial monitoring plans. Data quality and compliance with the protocol is assessed throughout the trial by central monitoring and site visits where required. The trial will undergo periodic audits by the trial's unit quality assurance team and the trial sponsor.

All reportable Serious Adverse Events are sent directly to the trial's unit and assessed by the Chief Investigator and reported as part of annual reports.

For peer review only - http://bmjopen.bmj.com/site/about/guidelines.xhtml

Confidentiality: Data obtained as a result of this study is considered confidential and confidentiality is ensured by utilising identification code numbers to correspond to treatment data.

Flagging with NHS Digital: Trial participation ends after 60 months with no further post-trial care (beyond standard practice). However, all women recruited to the study in the UK will be 'flagged' after discharge through the Data Linkage and Extract Service at NHS Digital to maximise outcome data collection and enable long-term follow-up data (greater than five years) to be reported.

Statistics

Sample Size: Among women who have adjuvant therapy plus axillary treatment, 2% have axillary recurrence within five years[11]. A total of 1700 women is required for analysis (850 in each group) in order to detect a non-inferiority margin of two percentage points for the risk difference using the Miettinen-Nurminen score interval[12], with 80% power and 2.5% one-sided alpha. Allowing for up to 10% non-compliance with treatment allocation and non-collection of primary outcome data, the target sample size to be randomised is 1900.

The magnitude of the non-inferiority margin was determined following consultation with relevant breast cancer consumer groups, lay people, breast surgeons and oncologists. There was consensus across all groups that an absolute increase in axillary recurrence of up to 2% is an acceptable trade-off for the increase in arm morbidity associated with axillary treatment.

Statistical Analysis: Analysis will be at the end of the five years follow-up of the last recruited participant. A detailed statistical analysis plan (SAP) will be developed by the trial statisticians in consultation with the Trial Management Group, and agreed with the Trial Steering Committee (TSC) before database lock and unblinding of data.

Descriptive statistics of demographic and clinical measures will be used to assess balance between the randomised arms at baseline. Continuous variables will be summarised in terms of the mean, standard deviation, median, lower and upper quartiles, minimum, maximum and number of observations. Categorical variables will be summarised in terms of frequency counts and percentages.

The primary approach for between-group comparative analyses of primary and secondary endpoints will be per protocol, and we will present point estimates and 95% confidence intervals. Sensitivity analysis where participants are analysed as randomised, regardless of compliance with allocation, will also be conducted for the primary outcome.

The primary analysis will assess whether adjuvant therapy alone is non-inferior to adjuvant therapy plus axillary treatment based on the primary endpoint of axillary recurrence within five years. The risk difference will be estimated using a mixed effects generalised linear model for binary outcome, with treatment arm and minimisation variables as fixed effects and recruitment site a random effect. Non-inferiority of adjuvant therapy alone compared with adjuvant therapy plus axillary treatment will be accepted if the upper boundary of the Miettinen-Nurminen two-sided 95% confidence limit for estimated difference in axillary recurrence is no greater than 2%.

We will conduct subgroup analyses of axillary recurrence and arm morbidity by fitting appropriate interaction terms in the regression models, according to the following variables

For peer review only - http://bmjopen.bmj.com/site/about/guidelines.xhtml

measured at baseline: number of sentinel node macrometastases; age; type of breast surgery; estrogen receptor (ER) status; tumour grade; sentinel node assessment technique; extranodal invasion. We will present subgroup-specific effects and 95% confidence intervals, as well as interactions, 95% confidence intervals and p-values. Due to the limited power available to detect all except large interactions, these subgroup analyses will be regarded as exploratory and interpreted with due caution

Analyses for surgical complications following treatment of ANC will be of a descriptive nature.

Economic Analysis: The economic evaluation will be carried out from the perspective of the NHS over a 3-year and 5-year time horizon. A 3-year time horizon was adopted for the cost-utility analysis as it was anticipated, that by 3-years post-randomisation, any differences in QoL between the intervention and standard care participants which might have been present earlier will have disappeared. Costs will be based upon the costs of the randomised interventions received and on the use of subsequent care and services.

For each participant, all clinical events (i) relevant to management and consequences following the initial sentinel node biopsy (adjuvant therapy alone or adjuvant therapy plus axillary treatment) (ii) up to the end of follow-up (iii) which occur in the hospital setting, will be recorded. These include primary treatments, re-admissions for complications or recurrences and associated adverse events. Events deemed unrelated to the consequences of the sentinel node biopsy or axillary treatment will not be included. In addition to these clinical events, participants will be asked to recall the extent to which they accessed primary care (e.g. GP) or other non-hospital services (e.g. physiotherapy visits) for their breast cancer at 6, 12, 24, 36, 48 and 60 months post-randomisation. Information relating to healthcare resource use will be collected via the eCRF. Costs will be estimated using routine sources (e.g. NHS reference costs, PSSRU). On the basis of each participant's record and the costs collected from routine sources, a total hospital cost and a total non-hospital cost will be derived for each participant for the duration of follow-up. These total costs will be combined to estimate the total cost per participant from which the total average cost per randomised group will be calculated. The breast and sentinel node biopsy procedure, systemic treatment, radiotherapy to breast or chest wall will not be included in the determination of costs, because all participants will undergo these treatment.

1) Cost-effectiveness analysis based on the incremental cost per reduction in axillary recurrence. Mean costs for each randomised arm will be calculated as will mean axillary recurrence and will be presented as point estimates of mean incremental costs and effects (reduced axillary recurrences) and the incremental cost per reduction in axillary recurrence at 5-years post-randomisation.

2) Cost-utility analysis based on the incremental cost per QALY gained. QALYs will be based on the area under the curve approach[13] using responses to the EQ-5D-5L completed at scheduled time points; baseline, 3, 6, 12, 24, and 36 months post-randomisation. The total average QALYs accruing in the intervention and standard care groups will be analysed for significant differences, following allowances for mortality (if occurring). Both mean cost and QALYs will be presented for each randomised group and incremental mean costs and QALY calculated along with the incremental cost per QALY gained at 3-years post-randomisation.

For peer review only - http://bmjopen.bmj.com/site/about/guidelines.xhtml

Both deterministic and stochastic sensitivity analyses will be used to address any uncertainty in our estimation of costs, effects and cost-effectiveness. Any uncertainty in the results, based on the stochastic sensitivity analyses, will be illustrated using cost-effectiveness planes and cost-effectiveness acceptability curves. Costs and effects will be discounted at UK recommended rates[14] as they are being estimated beyond a 1-year time horizon.

Radiotherapy Quality Assurance (RT QA): The UK Radiotherapy Trials QA (RTTQA) Group will be responsible for implementing and coordinating the RT QA programme through a named contact for the trial. The programme includes pre-trial and on-trial activities and is outlined below:

Pre-trial QA - Prior to site activation:

- Facility questionnaire – Details of treatment technique, immobilisation, verification and dosimetry will be recorded.

- Dummy Run - All centres must submit a test participant case to the RTTQA group, for review, demonstrating planned radiotherapy treatment to the breast/chest wall, axilla and supraclavicular fossa. The outlining, planning and treatment of this participant case should reflect how the centre intends to plan and treat all participants recruited to POSNOC.

On-trial QA – Ongoing QA during trial recruitment:

- Retrospective individual case reviews – All centres must export radiotherapy plan data to the RTTQA Group for their first ten participants. Data will include participant history, CT data, structure set, plan and dose files. The retrospective review process should include at least three plans with radiotherapy to the nodal region.

- Ongoing data collection - Radiotherapy data related to the RT QA programme will be collected and stored by the RTTQA Group for all participants, regardless of study allocation. A random sample of plans will be reviewed from each site during the trial recruitment period.

The sites will be required to adhere to the POSNOC radiotherapy planning and delivery guidelines when treating participants in the study. The RTTQA Group will promote and monitor compliance to the guidelines for the duration of trial recruitment by systematically reviewing a sample of radiotherapy plans submitted by centres. Radiotherapy guideline deviations, non-compliance or areas of concern will be recorded and if required, discussed with the TMG and individual centres.

Patient and public involvement: As part of the development of the project and protocol, women from the Derby Breast Cancer Support Group, Bosom Buddies (Guildford Breast Cancer Support Group) and Independent Cancer Patients' Voice (ICPV) have commented at all stages on the design and acceptability of the study. The Breast Cancer Care and Lymphoedema Support Network have had the opportunity to contribute to this protocol.

Independent Cancer Patients' Voice is an independent patient advocate group (http://independentcancerpatientsvoice.org.uk/). Two members of ICPV will provide a patient perspective throughout the duration of the study.

For peer review only - http://bmjopen.bmj.com/site/about/guidelines.xhtml

Ethics and dissemination

The study has been approved by the National Research Ethics Service (NRES) Committee East Midlands – Nottingham 2 (REC reference: 13/EM/0459).

The dissemination of the study results will be via a study report and research papers for publication in peer reviewed journals, and presentation at relevant conferences. Reporting will be in compliance with CONSORT recommendations. Publication of the results will be based on outcomes at 5-years following the last recruited participant.

A summary of the results will be made available to participants through a newsletter (unless they state they do not wish to receive this), and will also be publicised through Independent Cancer Patients' Voice, Cancer Research UK, Breast Cancer Now and Lymphoedema Support Network.

Trial status: Recruiting. On 09 June 2021, 1866 patients have been included.

Data sharing plan: Requests for data collected for POSNOC will be considered by the TMG and NCTU Data Sharing review panel.

Additional Documentation: The current full trial protocol (v 6.0 26 March 2020), details of previous protocol amendments, copies of current PIL and informed consent forms, oversight committee charters and a full list of participating sites can be found at www.posnoc.co.uk.

Author contributions

Conceptualization and writing original protocol: AG conceived the study and prepared the first draft of the original protocol.

Funding acquisition (UK and ANZ): AG, BM, LF, LD, MWR, DD, RC, AF, RGN, VJ, DW, MC, DW, VK, IE, PF

Trial design and methodology: All authors contributed to study design and methodology (AG, GBM, LF, LD, MWR, DD, RC, AF, RGN, VJ, DW, MC, DW, VK, IE, PF, SS, KM, AAM, WT, LV, TH, HB, RH, ML, DM, ZN, PS, AC, EM).

Statistics: AF, RGN, AM, WT

Review and editing: All authors read, critically revised and approved the manuscript before submission (AG, GBM, LF, LD, MWR, DD, RC, AF, RGN, VJ, DW, MC, DW, VK, IE, PF, SS, KM, AAM, WT, LV, TH, HB, RH, ML, DM, ZN, PS, AC, EM).

Radiotherapy QA: DD, DM, ZN, PS, AC, EM

Trial management and co-ordination: All authors are current or past TMG members and contributed to trial management and co-ordination (AG, GBM, LF, LD, MWR, DD, RC, AF, RGN, VJ, DW, MC, DW, VK, IE, PF, SS, KM, AAM, WT, LV, TH, HB, RH, ML, DM, ZN, PS, AC, EM).

For peer review only - http://bmjopen.bmj.com/site/about/guidelines.xhtml

Footnotes

Trial Management Group members: Amit Goyal (Chief Investigator and Chair), Shabina Sadiq, Mickey Lewis, Rachel Haines, Alan Montgomery, Wei Tan, G Bruce Mann, Kathryn Monson, Valerie Jenkins, Patricia Fairbrother, David Dodwell, Tara Homer, Heath Badger, Zohal Nabi, Romaana Mir, Elizabeth Miles, Luke Vale, Malcolm W Reed, Lesley Fallowfield, Robert Coleman, Vaughan Keeley, Ian Ellis, Daniel Davis, Teresa Grieve (past members: Lelia Duley, Diane Whitham, Margaret Childs, Apostolos Fakis, Robert G Newcombe, David Whynes, Eleanor Mitchell, Rebecca Haydock, Clare Brittain, Clare Upton, Sarah Craig)

Nottingham Clinical Trials Unit staff: Alan Montgomery, Rachel Haines, Shabina Sadiq, Wei Tan, Mickey Lewis, Aisha Shafayat, Charlotte Gidman, Mara Ozolins, Lelia Duley, Diane Whitham, Margaret Childs, Eleanor Mitchell, Rebecca Haydock, Clare Brittain, Clare Upton, Sarah Craig

Patient Reported Outcomes staff (Sussex Health Outcomes Research & Education in Cancer (SHORE-C)): Kathryn Monson, Valerie Jenkins, Lesley Fallowfield

Health Economics staff (Newcastle University): Tara Homer, Luke Vale (past staff: Laura Ternent)

Breast Cancer Trials Australia and New Zealand staff: Heath Badger, Bruce Mann, Nicole Francis, Flonda Probert, Lisa Paksec, Rose Lucas (past staff: Annette Dempsey, Rochelle Thornton)

Radiotherapy Quality Assurance team: Daniel Megias, Zohal Nabi, Preetinder Singh, Andrei Caraman, Elizabeth Miles, Romaana Mir, Roeum Butt, Hannah Price

Independent Trial Steering Committee members: Alastair Thompson (Chair), Alistair Ring, Nisha Sharma, Peter Barrett Lee, Julie Wolfarth, Ian White, Ramsey Cutress (past members:

Independent Data Monitoring Committee members: Judith Bliss (chair), Daniel Rea, Peter Dubsky

Sponsor: The trial is sponsored by the University Hospitals of Derby and Burton NHS Foundation Trust (uhdb.sponsor@nhs.net)**.** Sponsor staff - Daniel Davis, Joanne Thornhill, Anne Shaw, Amy Farmer, Teresa Grieve

Acknowledgments: We thank the patients who are participating in this trial, and staff at the participating centres and the independent data monitoring committee and trial steering committee for overseeing the trial.

Funding: This project is funded by the National Institute for Health Research Health Technology Assessment Programme (project number 12/35/17). Australian and New Zealand participation is funded by National Health and Medical Research Council (NHMRC) (project grant 1083172).

For peer review only - http://bmjopen.bmj.com/site/about/guidelines.xhtml

Disclaimer: The views expressed are those of the authors and not necessarily those of the Health Technology Assessment Programme, NIHR, NHS or the Department of Health and Social Care.

Competing interests: LV was a member of the NIHR Health Technology Assessment Programme, Clinical Trials and Evaluation Panel from 2014-2018.

Figure 1. Trial flow chart

Reference List

1. Buchholz, T.A., et al., *Impact of systemic treatment on local control for patients with lymph node-negative breast cancer treated with breast-conservation therapy.* J.Clin.Oncol., 2001. **19**(8): p. 2240-2246.
2. Mansel, R.E., et al., *Results of a national training programme in sentinel lymph node biopsy for breast cancer.* Br J Surg, 2013. **100**(5): p. 654-61.
3. Khonji, N.I., et al., *Incidence of nodes in completion mastectomy specimens following breast conservation and axillary clearance.* Br.J.Surg., 2002. **89**(10): p. 1294-1295.
4. Giuliano, A.E., et al., *Locoregional recurrence after sentinel lymph node dissection with or without axillary dissection in patients with sentinel lymph node metastases: the American College of Surgeons Oncology Group Z0011 randomized trial.* Ann Surg, 2010. **252**(3): p. 426-32; discussion 432-3.
5. Azghadi, S., M. Daly, and J. Mayadev, *Practice Patterns of Radiation Field Design for Sentinel Lymph Node-Positive Early-Stage Breast Cancer.* Clin.Breast Cancer, 2016. **16**(5): p. 410-417.
6. Donker, M., et al., *Radiotherapy or surgery of the axilla after a positive sentinel node in breast cancer (EORTC 10981-22023 AMAROS): a randomised, multicentre, open-label, phase 3 non-inferiority trial.* Lancet Oncol, 2014. **15**(12): p. 1303-1310.
7. Armer, J.M., et al., *Predicting breast cancer-related lymphedema using self-reported symptoms.* Nurs.Res., 2003. **52**(6): p. 370-379.
8. Kennedy, C.A., et al., *Measurement properties of the QuickDASH (Disabilities of the Arm, Shoulder and Hand) outcome measure and cross-cultural adaptations of the QuickDASH: a systematic review.* Qual.Life Res., 2013.
9. Coster, S., K. Poole, and L.J. Fallowfield, *The validation of a quality of life scale to assess the impact of arm morbidity in breast cancer patients post-operatively.* Breast Cancer Res.Treat., 2001. **68**(3): p. 273-282.
10. Spielberger, C., *The Manual for the State-Trait Inventory.* Palo Alto, CA: Consulting Psychologists Press Inc., 1983.
11. Sanghani, M., E.M. Balk, and B. Cady, *Impact of axillary lymph node dissection on breast cancer outcome in clinically node negative patients: a systematic review and meta-analysis.* Cancer, 2009. **115**(8): p. 1613-20.
12. Miettinen, O. and M. Nurminen, *Comparative analysis of two rates.* Stat Med, 1985. **4**(2): p. 213-26.
13. Matthews, J.N., et al., *Analysis of serial measurements in medical research.* BMJ, 1990. **300**(6719): p. 230-235.
14. *National Institute for Health and Care Excellence (NICE): Guide to the methods of technology appraisal 2013.* https://www.nice.org.uk/article/pmg9, 2013.

For peer review only

For peer review only - http://bmjopen.bmj.com/site/about/guidelines.xhtml

Figure 1. Trial flow chart

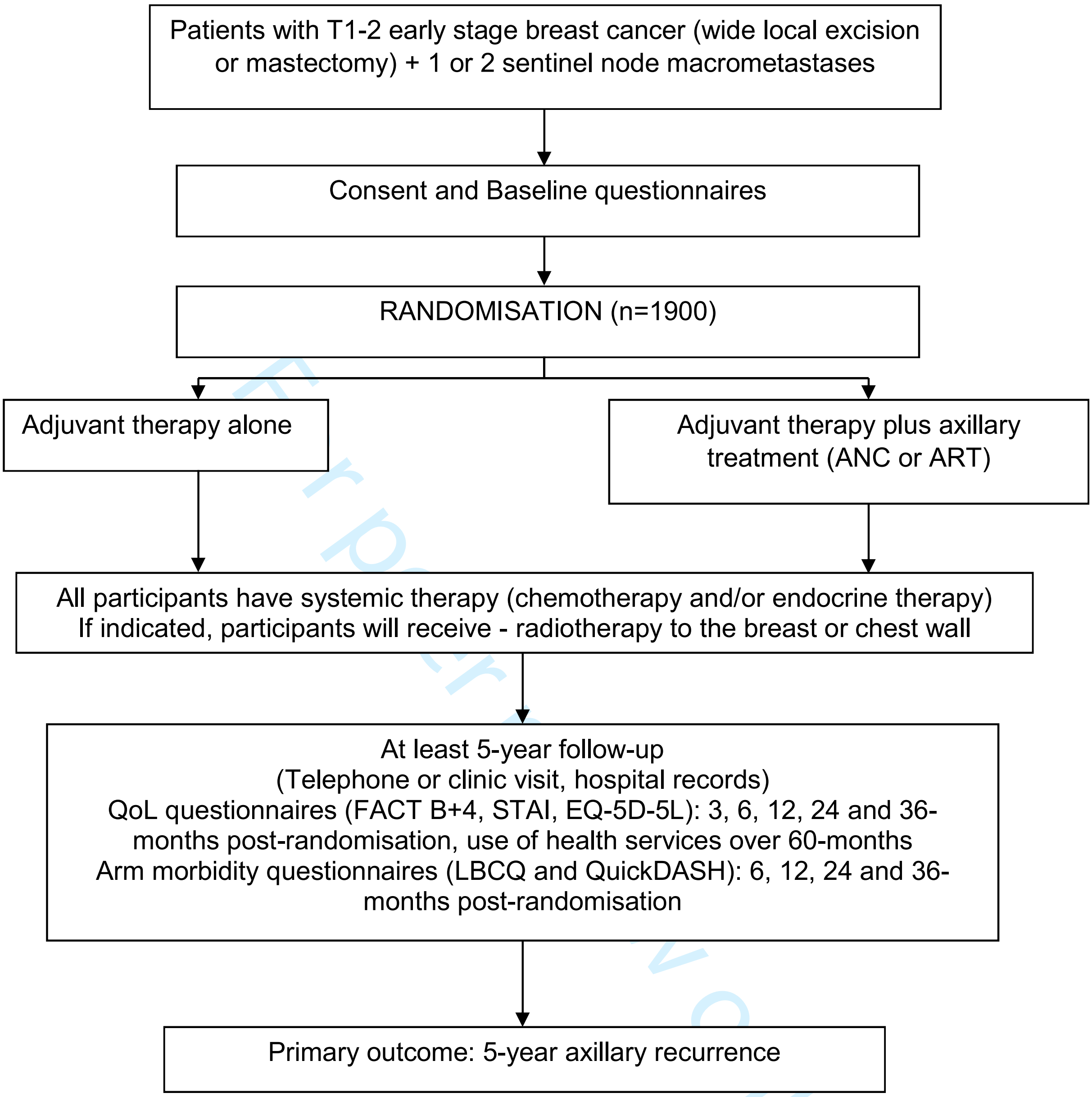

ANC, axillary node clearance; ART axillary radiotherapy; FACT B+4, Functional Assessment of Cancer Therapy – Breast +4; STAI, State-Trait Anxiety Inventory; LBCQ, Lymphoedema and Breast Cancer Questionnaire; QuickDASH, Disabilities of the Arm, Shoulder and Hand questionnaire.

For peer review only - http://bmjopen.bmj.com/site/about/guidelines.xhtml

SPIRIT

STANDARD PROTOCOL ITEMS: RECOMMENDATIONS FOR INTERVENTIONAL TRIALS

SPIRIT 2013 Checklist: Recommended items to address in a clinical trial protocol and related documents*

Section/item	Item No	Description	Addressed on page number
**Administrative information**			
Title	1	Descriptive title identifying the study design, population, interventions, and, if applicable, trial acronym	1
Trial registration	2a	Trial identifier and registry name. If not yet registered, name of intended registry	2
	2b	All items from the World Health Organization Trial Registration Data Set	throughout
Protocol version	3	Date and version identifier	13
Funding	4	Sources and types of financial, material, and other support	14
Roles and responsibilities	5a	Names, affiliations, and roles of protocol contributors	1, 13-14
	5b	Name and contact information for the trial sponsor	14
	5c	Role of study sponsor and funders, if any, in study design; collection, management, analysis, and interpretation of data; writing of the report; and the decision to submit the report for publication, including whether they will have ultimate authority over any of these activities	14
	5d	Composition, roles, and responsibilities of the coordinating centre, steering committee, endpoint adjudication committee, data management team, and other individuals or groups overseeing the trial, if applicable (see Item 21a for data monitoring committee)	13-14

For peer review only

Introduction

Background and rationale	6a	Description of research question and justification for undertaking the trial, including summary of relevant studies (published and unpublished) examining benefits and harms for each intervention	4
	6b	Explanation for choice of comparators	4
Objectives	7	Specific objectives or hypotheses	5
Trial design	8	Description of trial design including type of trial (eg, parallel group, crossover, factorial, single group), allocation ratio, and framework (eg, superiority, equivalence, noninferiority, exploratory)	5

Methods: Participants, interventions, and outcomes

Study setting	9	Description of study settings (eg, community clinic, academic hospital) and list of countries where data will be collected. Reference to where list of study sites can be obtained	5
Eligibility criteria	10	Inclusion and exclusion criteria for participants. If applicable, eligibility criteria for study centres and individuals who will perform the interventions (eg, surgeons, psychotherapists)	5-6
Interventions	11a	Interventions for each group with sufficient detail to allow replication, including how and when they will be administered	6
	11b	Criteria for discontinuing or modifying allocated interventions for a given trial participant (eg, drug dose change in response to harms, participant request, or improving/worsening disease)	n/a
	11c	Strategies to improve adherence to intervention protocols, and any procedures for monitoring adherence (eg, drug tablet return, laboratory tests)	n/a
	11d	Relevant concomitant care and interventions that are permitted or prohibited during the trial	6
Outcomes	12	Primary, secondary, and other outcomes, including the specific measurement variable (eg, systolic blood pressure), analysis metric (eg, change from baseline, final value, time to event), method of aggregation (eg, median, proportion), and time point for each outcome. Explanation of the clinical relevance of chosen efficacy and harm outcomes is strongly recommended	6-7
Participant timeline	13	Time schedule of enrolment, interventions (including any run-ins and washouts), assessments, and visits for participants. A schematic diagram is highly recommended (see Figure)	Figure 1, Table 1

For peer review only - http://bmjopen.bmj.com/site/about/guidelines.xhtml

Sample size	14	Estimated number of participants needed to achieve study objectives and how it was determined, including clinical and statistical assumptions supporting any sample size calculations	10
Recruitment	15	Strategies for achieving adequate participant enrolment to reach target sample size	9
**Methods: Assignment of interventions (for controlled trials)**			
Allocation:			
Sequence generation	16a	Method of generating the allocation sequence (eg, computer-generated random numbers), and list of any factors for stratification. To reduce predictability of a random sequence, details of any planned restriction (eg, blocking) should be provided in a separate document that is unavailable to those who enrol participants or assign interventions	5
Allocation concealment mechanism	16b	Mechanism of implementing the allocation sequence (eg, central telephone; sequentially numbered, opaque, sealed envelopes), describing any steps to conceal the sequence until interventions are assigned	5
Implementation	16c	Who will generate the allocation sequence, who will enrol participants, and who will assign participants to interventions	9
Blinding (masking)	17a	Who will be blinded after assignment to interventions (eg, trial participants, care providers, outcome assessors, data analysts), and how	n/a
	17b	If blinded, circumstances under which unblinding is permissible, and procedure for revealing a participant's allocated intervention during the trial	n/a
**Methods: Data collection, management, and analysis**			
Data collection methods	18a	Plans for assessment and collection of outcome, baseline, and other trial data, including any related processes to promote data quality (eg, duplicate measurements, training of assessors) and a description of study instruments (eg, questionnaires, laboratory tests) along with their reliability and validity, if known. Reference to where data collection forms can be found, if not in the protocol	9-10
	18b	Plans to promote participant retention and complete follow-up, including list of any outcome data to be collected for participants who discontinue or deviate from intervention protocols	9-10

For peer review only - http://bmjopen.bmj.com/site/about/guidelines.xhtml

Data management	19	Plans for data entry, coding, security, and storage, including any related processes to promote data quality (eg, double data entry; range checks for data values). Reference to where details of data management procedures can be found, if not in the protocol	9-10
Statistical methods	20a	Statistical methods for analysing primary and secondary outcomes. Reference to where other details of the statistical analysis plan can be found, if not in the protocol	10-12
	20b	Methods for any additional analyses (eg, subgroup and adjusted analyses)	10-12
	20c	Definition of analysis population relating to protocol non-adherence (eg, as randomised analysis), and any statistical methods to handle missing data (eg, multiple imputation)	10-12
**Methods: Monitoring**			
Data monitoring	21a	Composition of data monitoring committee (DMC); summary of its role and reporting structure; statement of whether it is independent from the sponsor and competing interests; and reference to where further details about its charter can be found, if not in the protocol. Alternatively, an explanation of why a DMC is not needed	14
	21b	Description of any interim analyses and stopping guidelines, including who will have access to these interim results and make the final decision to terminate the trial	n/a
Harms	22	Plans for collecting, assessing, reporting, and managing solicited and spontaneously reported adverse events and other unintended effects of trial interventions or trial conduct	9
Auditing	23	Frequency and procedures for auditing trial conduct, if any, and whether the process will be independent from investigators and the sponsor	9
**Ethics and dissemination**			
Research ethics approval	24	Plans for seeking research ethics committee/institutional review board (REC/IRB) approval	13
Protocol amendments	25	Plans for communicating important protocol modifications (eg, changes to eligibility criteria, outcomes, analyses) to relevant parties (eg, investigators, REC/IRBs, trial participants, trial registries, journals, regulators)	13

Consent or assent	26a	Who will obtain informed consent or assent from potential trial participants or authorised surrogates, and how (see Item 32)	5
	26b	Additional consent provisions for collection and use of participant data and biological specimens in ancillary studies, if applicable	n/a
Confidentiality	27	How personal information about potential and enrolled participants will be collected, shared, and maintained in order to protect confidentiality before, during, and after the trial	10
Declaration of interests	28	Financial and other competing interests for principal investigators for the overall trial and each study site	14
Access to data	29	Statement of who will have access to the final trial dataset, and disclosure of contractual agreements that limit such access for investigators	13
Ancillary and post-trial care	30	Provisions, if any, for ancillary and post-trial care, and for compensation to those who suffer harm from trial participation	13
Dissemination policy	31a	Plans for investigators and sponsor to communicate trial results to participants, healthcare professionals, the public, and other relevant groups (eg, via publication, reporting in results databases, or other data sharing arrangements), including any publication restrictions	13
	31b	Authorship eligibility guidelines and any intended use of professional writers	13
	31c	Plans, if any, for granting public access to the full protocol, participant-level dataset, and statistical code	13
**Appendices**			
Informed consent materials	32	Model consent form and other related documentation given to participants and authorised surrogates	13
Biological specimens	33	Plans for collection, laboratory evaluation, and storage of biological specimens for genetic or molecular analysis in the current trial and for future use in ancillary studies, if applicable	n/a

*It is strongly recommended that this checklist be read in conjunction with the SPIRIT 2013 Explanation & Elaboration for important clarification on the items. Amendments to the protocol should be tracked and dated. The SPIRIT checklist is copyrighted by the SPIRIT Group under the Creative Commons "Attribution-NonCommercial-NoDerivs 3.0 Unported" license.

For peer review only
